# Supplementary material for: Twenty years of change in benthic communities across the Belizean Barrier Reef
Source: PLoS One. 2022 Jan 18;17(1):e0249155. doi: 10.1371/journal.pone.0249155 (PMC8765652; doi:10.1371/journal.pone.0249155)
Supplement: S1 File — (DOCX) [file pone.0249155.s001.docx]

**Supporting Information for Alves et al. 2021**

Alves, C., A. Valdivia, R.B. Aronson, N. Bood, K. Castillo, C. Cox, C. Fieseler, Z. Locklear, M. McField, L. Mudge, J. Umbanhowar, and J.F. Bruno. 2021. Twenty years of change in benthic communities across the Belizean Barrier Reef. PLOS ONE

**S1 Table. List of benthic categories grouped for analysis.** Grouping was necessary because not all benthic categories were consistently scored every survey year.

|  | **Benthic group** | **Lowest taxa identified** | **Grouping** |
| --- | --- | --- | --- |
|  | Hard coral | *Acropora cervicornis* | *Acropora* spp. |
|  |  | *Acropora palmata* | *Acropora* spp. |
|  |  | *Acropora prolifera* | *Acropora* spp. |
|  |  | *Acropora* sp. | *Acropora* spp. |
|  |  | *Agaricia agaricites* complex | *Agaricia agaricites* |
|  |  | *Agaricia fragilis* | Other coral species |
|  |  | *Agaricia grahamae* | Other coral species |
|  |  | *Agaricia humilis* | Other coral species |
|  |  | *Agaricia lamarcki* | Other coral species |
|  |  | *Agaricia tenuifolia* | *Agaricia tenuifolia* |
|  |  | *Colpophyllia natans* | *Colpophyllia natans* |
|  |  | *Dendrogyra cylindrus* | *Dendrogyra cylindrus* |
|  |  | *Dichocoenia stokesi* | Other coral species |
|  |  | *Dichocoenia stokesii* | Other coral species |
|  |  | *Diploria clivosa* | *Diploria/Pseudodiploria* spp. |
|  |  | *Diploria labyrinthiformis* | *Diploria/Pseudodiploria* spp. |
|  |  | *Diploria strigosa* | *Diploria/Pseudodiploria* spp. |
|  |  | *Eusmilia fastigiata* | Other coral species |
|  |  | *Favia fragum* | Other coral species |
|  |  | *Isophyllia rigida* | Other coral species |
|  |  | *Isophyllia sinuosa* | Other coral species |
|  |  | *Leptoseris cucullata* | Other coral species |
|  |  | *Madracis auretenra* | Other coral species |
|  |  | *Madracis decactis* | Other coral species |
|  |  | *Madracis mirabilis* | Other coral species |
|  |  | *Manicina areolata* | Other coral species |
|  |  | *Meandrina meandrites* | Other coral species |
|  |  | *Millepora alcicornis* | Other coral species |
|  |  | *Millepora complenata* | Other coral species |
|  |  | *Millepora* sp. | Other coral species |
|  |  | *Montastrea cavernosa* | *Montastrea cavernosa* |
|  |  | *Mussa angulosa* | Other coral species |
|  |  | *Mycetophyllia aliciae* | Other coral species |
|  |  | *Mycetophyllia danaana* | Other coral species |
|  |  | *Mycetophyllia ferox* | Other coral species |
|  |  | *Mycetophyllia lamarckiana* | Other coral species |
|  |  | *Mycetophyllia* sp. | Other coral species |
|  |  | *Orbicella annularis* | *Orbicella* spp. |
|  |  | *Orbicella faveolata* | *Orbicella* spp. |
|  |  | *Orbicella franksi* | *Orbicella* spp. |
|  |  | *Porites astreoides* | *Porites astreoides* |
|  |  | *Porites colonensis* | *Porites* spp. |
|  |  | *Porites divaricata* | *Porites* spp. |
|  |  | *Porites furcata* | *Porites* spp. |
|  |  | *Porites porites* | *Porites* spp. |
|  |  | *Pseudodiploria clivosa* | *Diploria/Pseudodiploria* spp. |
|  |  | *Pseudodiploria strigosa* | *Diploria/Pseudodiploria* spp. |
|  |  | *Scleractinia* | Other coral species |
|  |  | *Scolymia cubensis* | Other coral species |
|  |  | *Scolymia* sp. | Other coral species |
|  |  | *Siderastrea radians* | *Siderastrea* spp. |
|  |  | *Siderastrea siderea* | *Siderastrea* spp. |
|  |  | *Siderastrea* sp. | *Siderastrea* spp. |
|  |  | *Solenastrea bournoni* | Other coral species |
|  |  | *Stephanocoenia intersepta* | Other coral species |
|  | Macroalgae | *Amphiroa* sp*.* | Calcareous algae |
|  |  | Branching coralline | Calcareous algae |
|  |  | Branching coralline antillarum | Calcareous algae |
|  |  | Calcareous algae | Calcareous algae |
|  |  | *Dictyota* sp. | Fleshy macroalgae |
|  |  | Erect rhodophyta | Corticated algae |
|  |  | Fleshy macroalgae | Fleshy macroalgae |
|  |  | *Galaxaura* sp. | Corticated algae |
|  |  | *Halimeda* sp*.* | Calcareous algae |
|  |  | *Lobophora* sp. | Corticated algae |
|  |  | Macroalgae | Fleshy macroalgae |
|  |  | *Padina* sp. | Corticated algae |
|  |  | *Venticaria* sp. | Corticated algae |
|  |  | *Wrangelia* sp. | Fleshy macroalgae |
|  | Crustose algae - Turf - Bare | Bare substrate | Crustose coralline - Turf - Bare |
|  |  | Crustose turf bare | Crustose coralline - Turf - Bare |
|  |  | Crustose coralline algae | Crustose coralline - Turf - Bare |
|  |  | Dead substrate | Crustose coralline - Turf - Bare |
|  |  | Encrusting coralline | Crustose coralline - Turf - Bare |
|  |  | Turf algae | Crustose coralline - Turf - Bare |
|  | Soft coral | Gorgonian | Gorgonian |
|  | Sponge | Sponge | Sponge |
|  | Other invertebrates | Anemone | Other invertebrates |
|  |  | Annelida | Other invertebrates |
|  |  | Ascidians | Other invertebrates |
|  |  | Barnacle | Other invertebrates |
|  |  | Bivalve | Other invertebrates |
|  |  | Black octocorals | Other invertebrates |
|  |  | Corallimorph | Other invertebrates |
|  |  | Hydroid | Other invertebrates |
|  |  | Mat tunicate | Other invertebrates |
|  |  | Nudibranch | Other invertebrates |
|  |  | Sea cucumber | Other invertebrates |
|  |  | Tunicate | Other invertebrates |
|  |  | Zoanthid | Other invertebrates |
|  | Bacterial mat | Cyanobacteria | Cyanobacteria |
|  | Other | Equipment | Other |
|  |  | Sand sediment | Other |
|  |  | Rubble | Other |
|  |  | Unknown | Other |
|  |  |  |  |

**S2 Table. Variation in three metrics of thermal stress anomaly (TSA) across years and sites.**Darker color represents higher values within each TSA metric. TSA is the weekly SST minus the maximum weekly climatological SST. The frequency of TSA (TSA_Freq) is the number of instances TSA is equal to or greater than 1 degree Celsius (°C) during the 52 weeks preceding a reef survey. The frequency of historical TSA (TSA_Freq_hist) is the number of times that TSA was ≥1 °C since the beginning of the dataset (i.e., 1982). The frequency of TSA between survey years (TSA_Freq_btw_surveys) is the number of instances that TSA was ≥1 °C since the previous survey year.

| **Site** | **TSA_Freq** | | | | | **TSA_Freq_hist** | | | | | **TSA_Freq_btw_surveys** | | | | |
| --- | --- | --- | --- | --- | --- | --- | --- | --- | --- | --- | --- | --- | --- | --- | --- |
|  | 1997 | 1999 | 2005 | 2009 | 2016 | 1997 | 1999 | 2005 | 2009 | 2016 | 1997 | 1999 | 2005 | 2009 | 2016 |
| Alligator | 1 | 3 | 1 | 5 | 2 | 9 | 15 | 22 | 33 | 52 | 4 | 3 | 5 | 6 | 14 |
| Bacalar Chico | 2 | 4 | 2 | 1 | 5 | 14 | 19 | 25 | 31 | 54 | 7 | 4 | 5 | 3 | 20 |
| Calabash | 0 | 1 | 0 | 4 | 3 | 24 | 25 | 34 | 42 | 54 | 9 | 1 | 4 | 5 | 10 |
| Gallows Reef | 1 | 2 | 1 | 2 | 0 | 16 | 20 | 24 | 37 | 57 | 6 | 2 | 4 | 5 | 16 |
| Goffs Caye | 0 | 3 | 1 | 4 | 1 | 11 | 15 | 19 | 30 | 43 | 4 | 3 | 3 | 6 | 11 |
| Halfmoon Caye | 1 | 2 | 1 | 1 | 1 | 6 | 8 | 18 | 28 | 47 | 2 | 2 | 6 | 6 | 15 |
| Hol Chan | 1 | 2 | 2 | 3 | 3 | 19 | 22 | 28 | 39 | 53 | 6 | 2 | 6 | 6 | 11 |
| Mexico Rocks | 0 | 3 | 3 | 4 | 0 | 7 | 12 | 22 | 31 | 40 | 4 | 3 | 9 | 5 | 5 |
| Middle Caye | 0 | 3 | 1 | 1 | 2 | 13 | 16 | 26 | 37 | 60 | 4 | 3 | 7 | 6 | 20 |
| Nicholas | 1 | 5 | 0 | 1 | 2 | 14 | 20 | 28 | 39 | 69 | 6 | 5 | 6 | 6 | 25 |
| Pompian | 1 | 5 | 0 | 3 | 2 | 19 | 25 | 31 | 43 | 67 | 7 | 5 | 3 | 7 | 21 |
| South of Middle Caye | 0 | 4 | 3 | 1 | 2 | 9 | 13 | 24 | 36 | 59 | 3 | 4 | 8 | 8 | 20 |
| South Water | 1 | 3 | 0 | 0 | 3 | 15 | 18 | 26 | 35 | 55 | 4 | 3 | 5 | 4 | 17 |
| Southwest Caye | 0 | 4 | 3 | 1 | 2 | 9 | 13 | 24 | 36 | 59 | 3 | 4 | 8 | 8 | 20 |
| Tacklebox | 1 | 5 | 4 | 4 | 5 | 27 | 33 | 45 | 56 | 72 | 7 | 5 | 11 | 5 | 13 |

**S3 Table. Summary of model comparisons via ANOVA for selecting the best TSA metric for each benthic group**. For each model, the model structure, Akaike Information Criterion (AIC), Bayesian Information Criterion (BIC), log-likelihood (LogLik), deviance, and Chi-square values are reported. All models had the following structure:

*{ Logit of Cover ~ Year + Protection_status + HII_50km + TSA_metric + (1|Site) }*

where: *TSA_metric* was *used TSA_Freq* for Model 1*,*

*TSA_Freq_Hist* for Model 2*,* and

*TSA_Freq_btw_surveys* for Model 3*.*

The model with TSA_Freq as TSA metric was selected for each benthic group.

| **Model Structure** | **AIC** | **BIC** | **LogLik** | **Deviance** | **Chisq** |  |
| --- | --- | --- | --- | --- | --- | --- |
| **Hard Coral** |  |  |  |  |  | |
| ***Model 1: TSA_Freq*** | 56.67 | 69.31 | -21.33 | 42.67 |  | |
| *Model 2: TSA_Freq_Hist* | 65.26 | 77.93 | -25.64 | 51.29 | 0.00 | |
| *Model 3: TSA_Freq_btw_surveys* | 64.92 | 77.57 | -25.46 | 50.92 | 0.36 | |
| **Macroalgae** |  |  |  |  |  | |
| ***Model 1: TSA_Freq*** | 63.99 | 76.64 | -25.00 | 49.99 |  | |
| *Model 2: TSA_Freq_Hist* | 67.45 | 80.10 | -26.73 | 53.45 | 0.00 | |
| *Model 3: TSA_Freq_btw_surveys* | 60.24 | 72.89 | -23.12 | 46.24 | 7.21 | |
| **CTB** |  |  |  |  |  | |
| ***Model 1: TSA_Freq*** | 98.43 | 111.08 | -42.22 | 84.43 |  | |
| *Model 2: TSA_Freq_Hist* | 100.19 | 112.83 | -43.09 | 86.19 | 0.00 | |
| *Model 3: TSA_Freq_btw_surveys* | 100.18 | 112.82 | -43.09 | 86.18 | 0.01 | |
| **Gorgonian** |  |  |  |  |  | |
| ***Model 1: TSA_Freq*** | 52.74 | 65.38 | -19.37 | 38.74 |  | |
| *Model 2: TSA_Freq_Hist* | 56.57 | 69.21 | -21.28 | 42.57 | 0.00 | |
| *Model 3: TSA_Freq_btw_surveys* | 59.55 | 72.20 | -22.78 | 45.55 | 0.00 | |
| **Sponge** |  |  |  |  |  | |
| ***Model 1: TSA_Freq*** | 93.28 | 105.93 | -39.64 | 79.28 |  | |
| *Model 2: TSA_Freq_Hist* | 96.77 | 109.41 | -41.38 | 82.77 | 0.00 | |
| *Model 3: TSA_Freq_btw_surveys* | 96.42 | 109.07 | -41.21 | 82.42 | 0.34 | |

**S4 Table. Estimated regression parameters for coral taxa coverage models.** Estimated regression parameters, standard errors, F-statistics, p-values, significance levels (Sig.), sign (positive or negative), and *marginal/conditional R^2^* from the final Bayesian generalized linear mixed model for each coral taxon are shown. Significance levels are: *** < 0.001; ** < 0.01, * < 0.05.

| **Coral Taxa /**  **Term** | **Estimate** | **Std. Error** | **Statistic** | **p-value** | **Sig.** | **Sign** |
| --- | --- | --- | --- | --- | --- | --- |
| ***Acropora* spp.** |  |  |  |  |  |  |
| (Intercept) | -3.614 | 0.018 | -196.441 | <0.001 | *** | neg |
| Year | -0.040 | 0.017 | -2.331 | 0.020 | * | neg |
| No Fishing vs. Fishing | 0.020 | 0.032 | 0.637 | 0.524 |  |  |
| HII_50km | 0.054 | 0.031 | 1.769 | 0.077 |  |  |
| TSA_Freq | -0.049 | 0.019 | -2.517 | 0.012 | * | neg |
| *Marginal R^2^/Conditional R^2^* | *0.055/0.120* |  |  |  |  |  |
| ***Agaricia agaricites*** |  |  |  |  |  |  |
| (Intercept) | -3.230 | 0.073 | -44.084 | <0.001 | *** | neg |
| Year | 0.009 | 0.038 | 0.228 | 0.820 |  |  |
| No Fishing vs. Fishing | 0.092 | 0.132 | 0.698 | 0.485 |  |  |
| HII_50km | 0.056 | 0.128 | 0.438 | 0.661 |  |  |
| TSA_Freq | 0.039 | 0.046 | 0.865 | 0.387 |  |  |
| *Marginal R^2^/Conditional R^2^* | *0.013/0.276* |  |  |  |  |  |
| ***Agaricia tenuifolia*** |  |  |  |  |  |  |
| (Intercept) | -3.439 | 0.065 | -52.818 | <0.001 | *** | neg |
| Year | 0.076 | 0.033 | 2.268 | 0.023 | * | pos |
| No Fishing vs. Fishing | -0.157 | 0.118 | -1.334 | 0.182 |  |  |
| HII_50km | 0.092 | 0.113 | 0.813 | 0.416 |  |  |
| TSA_Freq | -0.074 | 0.040 | -1.834 | 0.067 |  |  |
| *Marginal R^2^/Conditional R^2^* | *0.081/0.328* |  |  |  |  |  |
| ***Colpophyllia natans*** |  |  |  |  |  |  |
| (Intercept) | -3.574 | 0.035 | -101.309 | <0.001 | *** | neg |
| Year | -0.101 | 0.035 | -2.920 | 0.003 | ** | neg |
| No Fishing vs. Fishing | 0.045 | 0.060 | 0.737 | 0.461 |  |  |
| HII_50km | -0.028 | 0.059 | -0.479 | 0.632 |  |  |
| TSA_Freq | 0.019 | 0.039 | 0.485 | 0.628 |  |  |
| *Marginal R^2^/Conditional R^2^* | *0.030/0.083* |  |  |  |  |  |
| ***Dendrogyra cylindrus*** |  |  |  |  |  |  |
| (Intercept) | -3.652 | 0.013 | -285.877 | <0.001 | *** | neg |
| Year | 0.015 | 0.012 | 1.172 | 0.241 |  |  |
| No Fishing vs. Fishing | 0.002 | 0.022 | 0.107 | 0.915 |  |  |
| HII_50km | -0.019 | 0.021 | -0.874 | 0.382 |  |  |
| TSA_Freq | -0.015 | 0.014 | -1.046 | 0.295 |  |  |
| *Marginal R^2^/Conditional R^2^* | *0.015/0.071* |  |  |  |  |  |
| ***Diploria/Pseudodiploria* spp.** |  |  |  |  |  |  |
| (Intercept) | -3.485 | 0.046 | -76.370 | <0.001 | *** | neg |
| Year | 0.046 | 0.035 | 1.307 | 0.191 |  |  |
| No Fishing vs. Fishing | -0.004 | 0.080 | -0.045 | 0.964 |  |  |
| HII_50km | -0.141 | 0.078 | -1.811 | 0.070 |  |  |
| TSA_Freq | -0.046 | 0.041 | -1.129 | 0.259 |  |  |
| *Marginal R^2^/Conditional R^2^* | *0.049/0.160* |  |  |  |  |  |
| ***Montastrea cavernosa*** |  |  |  |  |  |  |
| (Intercept) | -3.484 | 0.027 | -128.655 | <0.001 | *** | neg |
| Year | 0.002 | 0.027 | 0.082 | 0.935 |  |  |
| No Fishing vs. Fishing | -0.036 | 0.046 | -0.783 | 0.433 |  |  |
| HII_50km | -0.001 | 0.045 | -0.017 | 0.986 |  |  |
| TSA_Freq | -0.063 | 0.031 | -2.029 | 0.043 | * | neg |
| *Marginal R^2^/Conditional R^2^* | *0.015/0.062* |  |  |  |  |  |
| ***Orbicella* spp.** |  |  |  |  |  |  |
| (Intercept) | -2.899 | 0.132 | -21.998 | <0.001 | *** | neg |
| Year | -0.719 | 0.061 | -11.787 | <0.001 | *** | neg |
| No Fishing vs. Fishing | 0.245 | 0.239 | 1.028 | 0.304 |  |  |
| HII_50km | 0.287 | 0.230 | 1.245 | 0.213 |  |  |
| TSA_Freq | -0.228 | 0.074 | -3.088 | 0.002 | ** | neg |
| *Marginal R^2^/Conditional R^2^* | *0.269/0.501* |  |  |  |  |  |
| ***Porites astreoides*** |  |  |  |  |  |  |
| (Intercept) | -3.310 | 0.048 | -69.626 | <0.001 | *** | neg |
| Year | 0.022 | 0.038 | 0.564 | 0.573 |  |  |
| No Fishing vs. Fishing | -0.114 | 0.083 | -1.368 | 0.171 |  |  |
| HII_50km | 0.174 | 0.081 | 2.152 | 0.031 | * | pos |
| TSA_Freq | -0.040 | 0.045 | -0.890 | 0.373 |  |  |
| *Marginal R^2^/Conditional R^2^* | *0.086/0.179* |  |  |  |  |  |
| ***Porites* spp.** |  |  |  |  |  |  |
| (Intercept) | -3.408 | 0.049 | -69.810 | <0.001 | *** | neg |
| Year | -0.033 | 0.038 | -0.866 | 0.386 |  |  |
| No Fishing vs. Fishing | -0.023 | 0.086 | -0.272 | 0.786 |  |  |
| HII_50km | 0.085 | 0.083 | 1.021 | 0.307 |  |  |
| TSA_Freq | -0.126 | 0.045 | -2.816 | 0.005 | ** | neg |
| *Marginal R^2^/Conditional R^2^* | *0.036/0.141* |  |  |  |  |  |
| ***Siderastrea* spp.** |  |  |  |  |  |  |
| (Intercept) | -3.482 | 0.044 | -78.501 | <0.001 | *** | neg |
| Year | 0.044 | 0.030 | 1.428 | 0.153 |  |  |
| No Fishing vs. Fishing | -0.003 | 0.079 | -0.032 | 0.974 |  |  |
| HII_50km | -0.007 | 0.076 | -0.098 | 0.922 |  |  |
| TSA_Freq | 0.049 | 0.036 | 1.362 | 0.173 |  |  |
| *Marginal R2/Conditional R2* | *0.012/0.161* |  |  |  |  |  |
| **Other coral species** |  |  |  |  |  |  |
| (Intercept) | -3.323 | 0.069 | -48.421 | <0.001 | *** | neg |
| Year | -0.342 | 0.044 | -7.758 | <0.001 | *** | neg |
| No Fishing vs. Fishing | 0.123 | 0.123 | 1.007 | 0.314 |  |  |
| HII_50km | 0.190 | 0.118 | 1.603 | 0.109 |  |  |
| TSA_Freq | -0.247 | 0.052 | -4.723 | 0.000 | *** | neg |
| *Marginal R2/Conditional R2* | *0.214/0.351* |  |  |  |  |  |

**S5 Table. Estimated regression parameters for models of the coverage of macroalgal functional groups.** Estimated regression parameters, standard errors, F-statistics, p-values, significance levels (Sig.), sign (positive or negative), and *marginal/conditional R^2^* from the final Bayesian generalized linear mixed models for each coral taxon are shown. Significance levels are: *** < 0.001; ** < 0.01; * < 0.05.

| Macroalgae group/Term | **Estimate** | **Std. Error** | | **Statistic** | | **p-value** | | **Sig.** | | **Sign** | |  |
| --- | --- | --- | --- | --- | --- | --- | --- | --- | --- | --- | --- | --- |
| **Calcareous macroalgae** | | |  | |  | |  | |  | |  | |
| (Intercept) | -3.158 | 0.134 | | -23.504 | | 0.000 | | *** | | neg | |  |
| Year | -0.400 | 0.064 | | -6.263 | | 0.000 | | *** | | neg | |  |
| No Fishing vs. Fishing | -0.303 | 0.229 | | -1.325 | | 0.185 | |  | | neg | |  |
| HII_50km | 0.238 | 0.234 | | 1.017 | | 0.309 | |  | | pos | |  |
| TSA_Freq | 0.247 | 0.077 | | 3.204 | | 0.001 | | ** | | pos | |  |
| *Marginal R^2^/Conditional R^2^* | *0.158/0.421* |  | |  | |  | |  | |  | |  |
| **Fleshy macroalgae** | | |  | |  | |  | |  | |  | |
| (Intercept) | -1.595 | 0.142 | | -11.239 | | 0.000 | | *** | | neg | |  |
| Year | 0.721 | 0.068 | | 10.611 | | 0.000 | | *** | | pos | |  |
| No Fishing vs. Fishing | 0.236 | 0.241 | | 0.976 | | 0.329 | |  | | pos | |  |
| HII_50km | 0.491 | 0.247 | | 1.987 | | 0.047 | | * | | pos | |  |
| TSA_Freq | 0.129 | 0.082 | | 1.568 | | 0.117 | |  | | pos | |  |
| *Marginal R^2^/Conditional R^2^* | *0.275/0.498* |  | |  | |  | |  | |  | |  |
| **Corticated macroalgae** | | |  | |  | |  | |  | |  | |
| (Intercept) | -3.127 | 0.091 | | -34.305 | | 0.000 | | *** | | neg | |  |
| Year | 1.182 | 0.060 | | 19.611 | | 0.000 | | *** | | pos | |  |
| No Fishing vs. Fishing | -0.095 | 0.153 | | -0.618 | | 0.537 | |  | | neg | |  |
| HII_50km | -0.239 | 0.156 | | -1.531 | | 0.126 | |  | | neg | |  |
| TSA_Freq | -0.330 | 0.072 | | -4.608 | | 0.000 | | *** | | neg | |  |
| *Marginal R^2^/Conditional R^2^* | *0.4970.583* |  | |  | |  | |  | |  | |  |

**
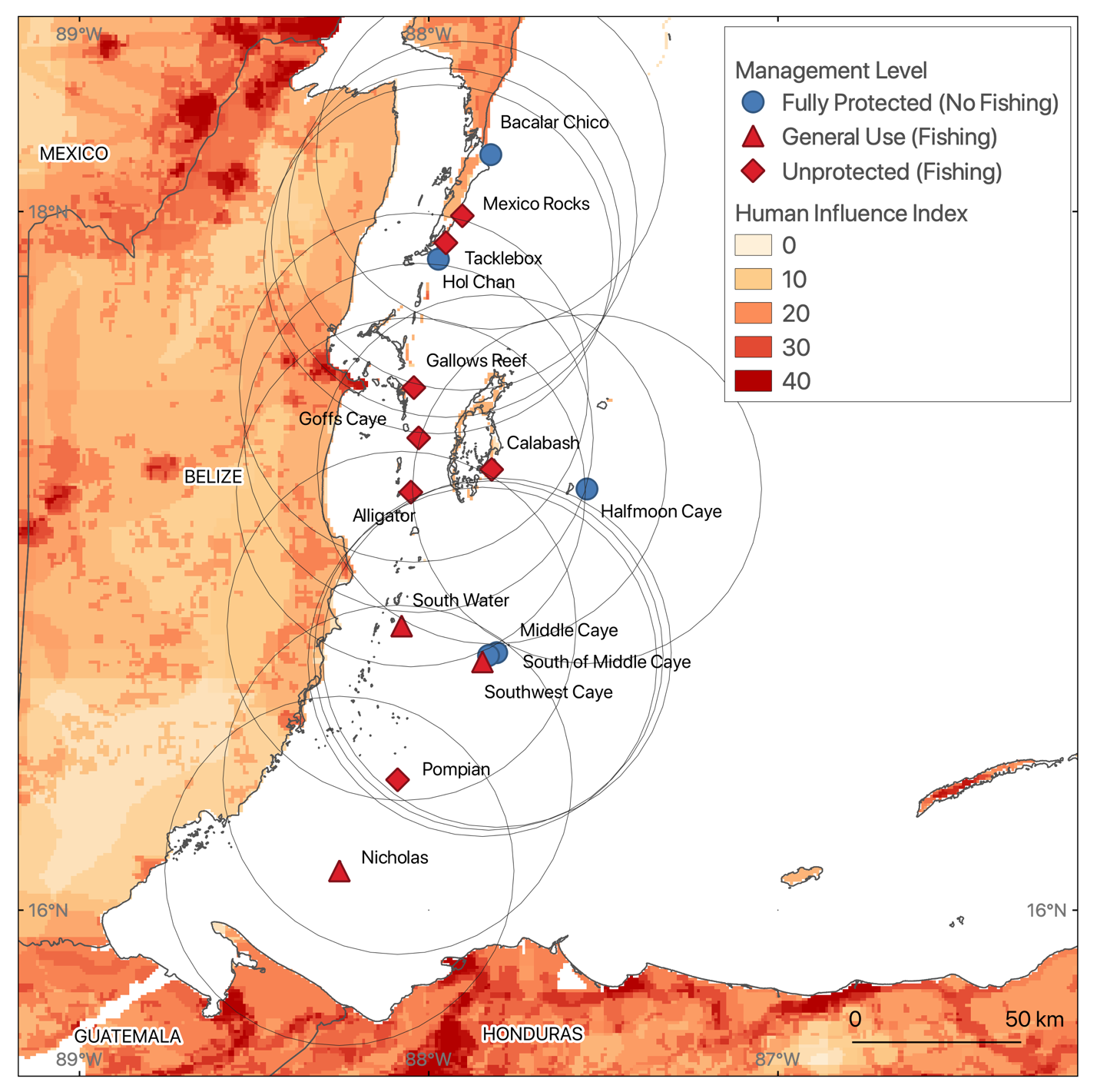
**

**S1 Fig. Human Influence Index (HII) estimated for 2005 across the landscape adjacent to the Belizean Barrier Reef.** Grey circles are 50-km radius buffers to calculate cumulative HHI for each site. GADM source: Database of Global Administrative Areas, Version 3.6 available at www.GADM.org.


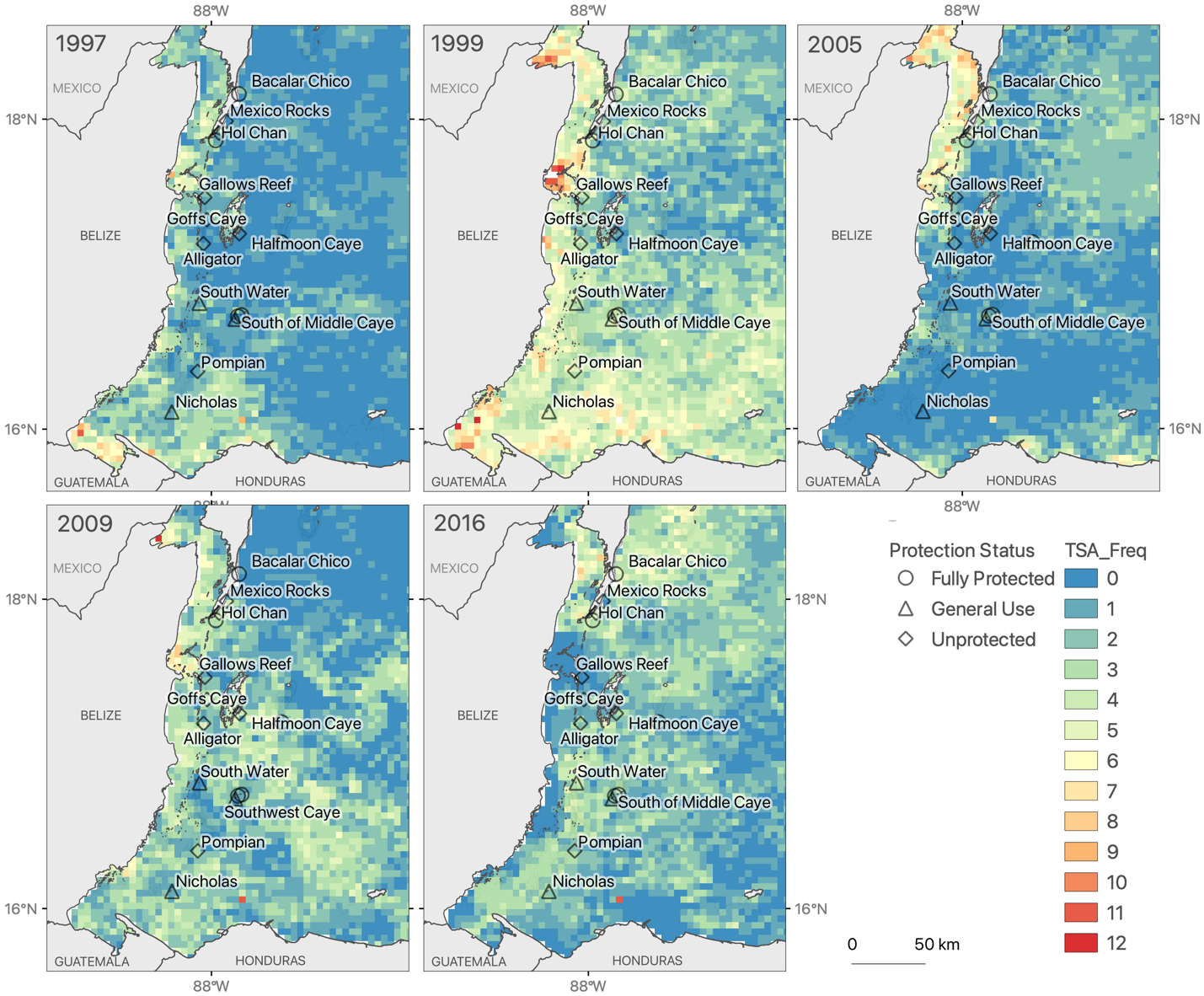


**S2 Fig. Frequency of thermal stress anomalies (TSA freq) for each survey year across the Belizean Barrier Reef.** See Table 2 for site-specific values.

**
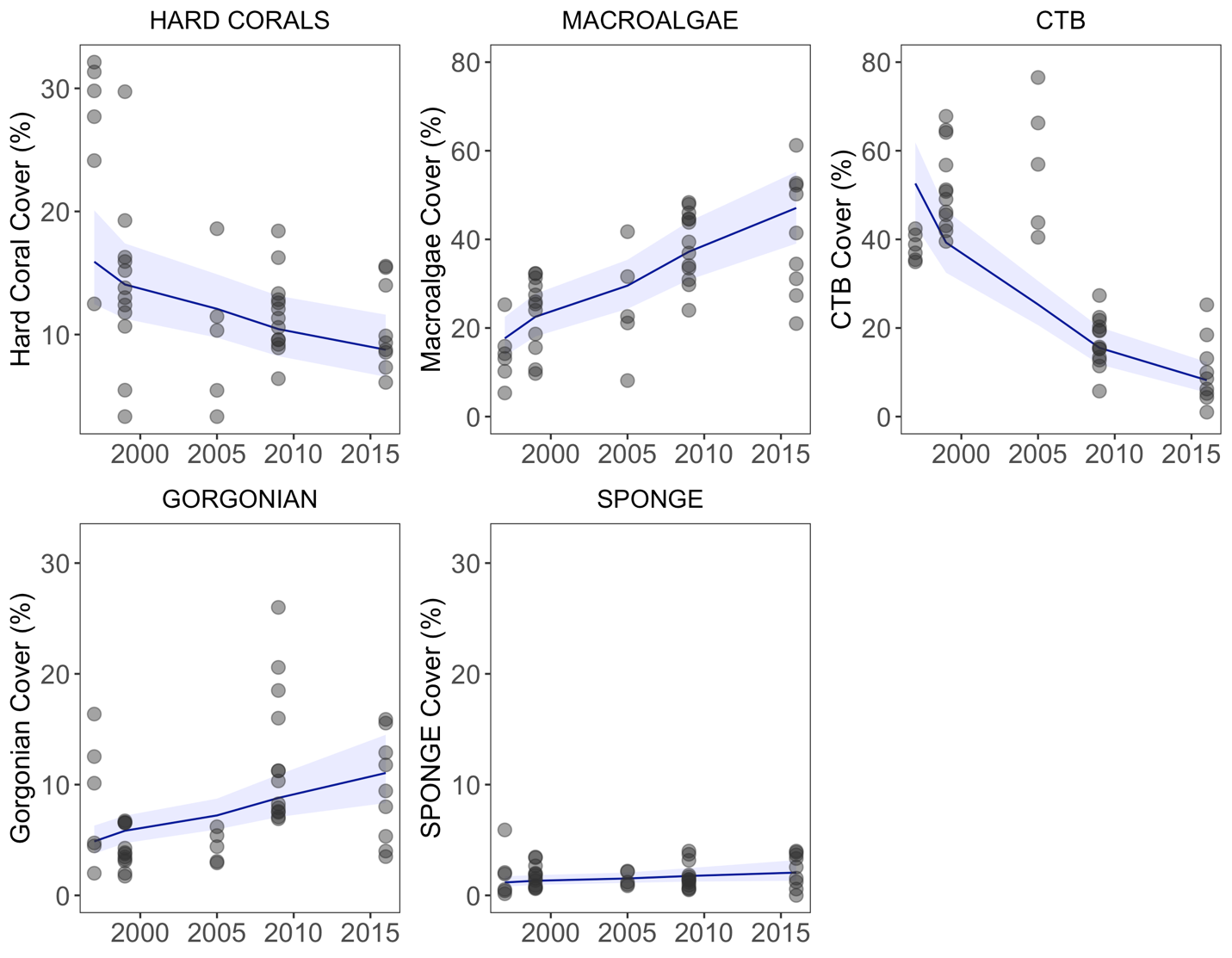
**

**S3 Fig. Relationship between predicted percent cover and year for each benthic category, accounting for several covariates**. Blue line is the fitted distribution from the BLMER models accounting for the effect of the covariates. Shading indicates 95% CI. Hard-coral, macroalgal, CTB, and gorgonian cover changed significantly from 1997 to 2016 (see Table 2 for model results).

**
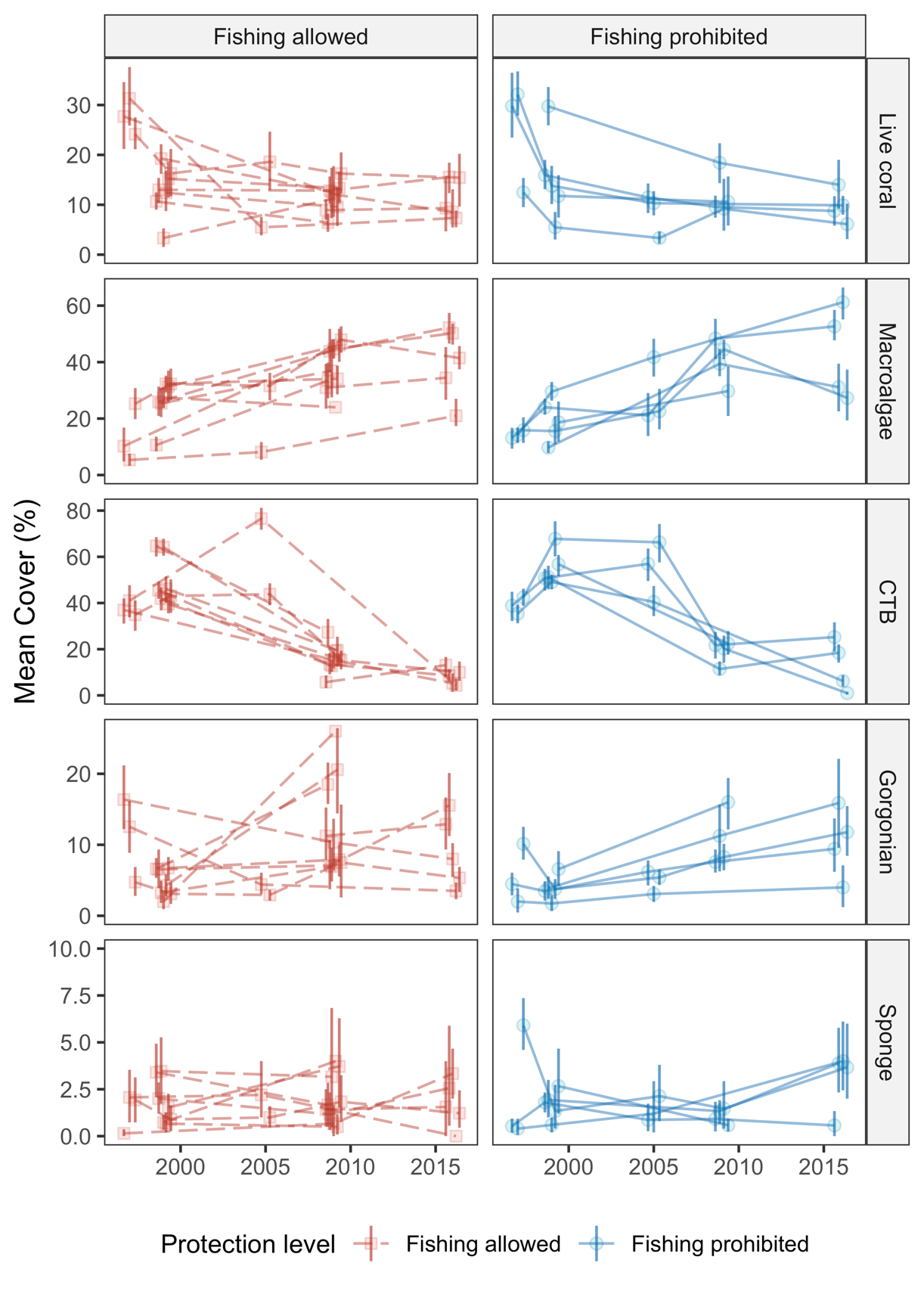
**

**S4 Fig. Site-level percent cover of five benthic categories over time, grouped by protection status**. Points are site means and error bars are 95% confidence intervals.
