# Supplementary material for: Twenty years of change in benthic communities across the Belizean Barrier Reef
Source: PLoS One. 2022 Jan 18;17(1):e0249155. doi: 10.1371/journal.pone.0249155 (PMC8765652; doi:10.1371/journal.pone.0249155)
Supplement: Attachment — Submitted filename: Alves_et_al_PLOS response letterR2R.pdf [file pone.0249155.s003.pdf]

THE UNIVERSITY of NORTH CAROLINA at CHAPEL HILL

College of Arts & Sciences
Department of Biology

DEPARTMENT OF BIOLOGY
COKER HALL
CAMPUS BOX 3280
CHAPEL HILL, NC 27599-3280 USA

PHONE: 919-962-2077
FAX: 919-962-1625
WEB: http://bio.unc.edu

June 10, 2021

Dear Dr. Coen,

Thank you very much for your detailed summary of the reviewers' concerns and suggestions, and for your own thoughtful perspective on Belize and our framing and interpretation of what's going on there.

We largely agree with everything you have said and have substantially revised the manuscript in accordance with your suggestions. We believe it is a much better manuscript now. Thank you for the time you put into it. It's unusual for an editor to provide clear instructions on what they want done and which of the reviewer suggestions they want authors to pay particular attention to. Below, we summarize what we've changed.

- Paper title: We believe the title reflects what was done and found, but we're open to suggestions from the editor on a better title.
- We have added substantial text describing HII; what it is, its value, etc. We also elaborate on the study design and our approach to hypothesis testing and inference, related limitations, etc.
- We have added text outlining the study limitations.
- We have added text to the Discussion describing the many changes to the mainland and cayes during the study. And thank you for the detailed information on the many aspects of the natural degradation of Belizean ecosystems. We also better explained our rationale for testing the role of local factors, MPAs, and ocean temperature.
- We have added text on the role of herbivores in driving benthic changes and on SCTLD. Although there isn't that much remaining coral to lose, SCTLD is impacting reefs across the region and this effect was not captured in our study. However, it is premature to declare that warming plays no role in enabling SCTLD. For example, it's only after the Caribbean sea warmed by ~1°C that SCTLD took off.
- We have added text on macroalgae-specific responses. Note the shoreline *Sargassum* issue isn't addressed because we are working on deeper, offshore reefs. The source of the shoreline *Sargassum* (observed in Belize and throughout the Caribbean) is thought to be sourced at a distance and surface / pelagic, not local to the benthic communities. We actually saw little *Sargassum* on the reefs; browns we're mainly *Dictyota* and *Lobophora*.

- Regarding concerns about inferences made from “a limited number of sites,” we added text to the Discussion on many other studies that have documented the same trends on other sites in Belize and across the region (literally hundreds of sites, all showing the same patterns).
- We have added text on survey protocols to the Methods. Regarding the comment, “Why were the transects only carried out in the summer?” the reason is that we teach during the school year and were only able to depart for ~3–4 week research trips during the summertime. While it is true that macroalgal community dynamics have a strong seasonal component, in our opinion it was best to control for this by repeating the surveys at the same time of year (late May). Thus, the answer to the comment, “How did you deal with seasonality of algae, etc.” is by standardizing the season we sampled.
- We have added substantial text on the limitations of Belize’s MPAs: lack of enforcement, etc. Note what we are reporting and basing the analysis on is the Belize Fisheries Dept’s management intention / classifications. Testing the effectiveness of this policy in protecting and restoring corals was the point of our study, corals, regardless of the implementation of the policy in terms of design, enforcement, etc. We elaborate on this in the added text. We also now include
- Regarding the length of Discussion: True. The Introduction and Discussion are very concise. We could have written a monograph, but purposely kept these two sections to a bare minimum in framing the study, describing our findings, and relating our results to prior work. Among us, we’ve written about these topics in many, many papers, and the literature includes thousands of papers describing coral decline, the putative causes, etc. We have expanded the sections requested by the reviewers while trying to keep the manuscript as concise as possible. In our opinion the results speak for themselves. The most valuable information is in the graphics describing the study design and observed trends
- In our view, the information in Table 1 is valuable, but we’d be happy to move it to the Appendix if the editor insists.

Below, we include our responses to the comments, in blue, from the reviewers.

We are confident that our substantial edits to this manuscript fit the scope of PLOS ONE and we are eager for your continued feedback.

Sincerely,

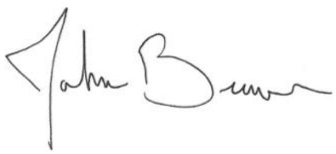

John F. Bruno, Ph.D., on behalf of all coauthors

Reviewer #1: Long data sets such as this are comparatively rare. While the original intent was not to publish a long term dataset when the project started, the data were collected for various reasons and it makes sense to present it as a long look back.

Reviewer #2: In this contribution, the authors address the long-running debate among coral reef ecologists of the relative importance of local vs. broad-scale stressors on reef health (here, as coral cover) with a time-series analysis of reef transects from sites in fished vs. MPA reefs across 15 sites on the BBR over 19 years. **This reviewer is in complete agreement that broad-scale stressors are what matters, particularly for Caribbean reefs** (although not in complete agreement with how the debate is framed in this paper, see below). I am generally supportive of publication of this work, particularly because standardized time-series data are valuable and rare. However, there are weaknesses in this paper that will probably not convince "the other side" in the debate (***not that this could occur, even with incontrovertible proof***), and I think the authors should consider revisions that better recognize the limitations in this study.

The biggest limitation is the lack of independent confirmation (validation) that MPAs were enforced during the study period. Figures indicate "fishing" and "no fishing" without anything to back this up. The easiest way to do this is to provide data on fish abundance or biomass, minimally parrotfish abundance or biomass, and then analyze the data relative to MPA effectiveness (as fish biomass). Unenforced MPAs are likely no different than fished areas, and Belize does not have a good record of MPA enforcement. Easy-access reefs (close to towns, villages, resorts) may be designated MPAs and highly overfished, while isolated reefs may not be designated as MPAs, but are lightly fished. If the authors have no data regarding MPA enforcement, then they need to make it very clear that they did not assess MPA effectiveness, and tone down their conclusions accordingly.

This is a fair point and perennial challenge to MPA efficacy / impact studies. Most MPAs in Belize (Cox et al. 2017), across the Caribbean (Valdivia et al. 2017), and globally (Gill et al. 2017) have minimal or no effect on fish biomass and other measures of efficacy. In Cox et al. (2017), we measured fish composition and biomass inside no-take reserves and in nearby control sites and found they were (largely) ineffective. We did not / could not quantify fishing activity in the no-take reserves. But there is existing information on enforcement, which we have now incorporated in the analysis and interpretation of our results (see Table S1, and table below for more details). The enforcement information comes from the Mesoamerican Eco-Audits conducted by the Healthy Reef Initiative in collaboration with the World Resources Institute and local partners across Belize, Guatemala, Honduras and Mexico. These eco-audits, conducted every two years since 2011, evaluate the degree of implementation of 28 recommended management actions (including enforcement) in coral reefs.

We addressed this concern in the original text of the submitted manuscript: "Like MPAs across the Caribbean (5), even the fully protected marine reserves in Belize have minimal effects on reef fish biomass and composition (5,46). This alone could explain the absence of an effect of

protection status on benthic community dynamics." And we now expand on it substantially in the Discussion section.

Marine Protected Area	Year	Marine Area (km^2)	2017 RZ Area (km^2)	Enforcement Level* 2020	Enforcement Level* 2016	Enforcement Level* 2014	Enforcement Level* 2011
Bacalar Chico MR	1996	34	15	Inadequate	Moderate	Moderate	Moderate
Hol Chan MR	1987	402	90	Good	Good	Good	Good
Halfmoon Caye NM	1982	39	39	Moderate	Good	Moderate	Moderate
Glovers Reef MR	1993	351	79	Moderate	Good	Moderate	Moderate
South Water Caye MR	1996	468	86	Moderate	Moderate	Inadequate	Inadequate

Good: Regular patrols, overall satisfactory compliance and ecological integrity is thought to be maintained,
Moderate: Regular patrols conducted, but limited poaching occurs, legal outcomes are insufficient, and ecological integrity is slightly impacted,
Inadequate: Irregular patrols conducted, poaching persists, legal outcomes are insufficient, ecological integrity is impacted, and local community feedback demonstrates a high level of concern

It is a fair criticism that one explanation for the lack of an MPA effect detected in our study is that these local protections were ineffective. On the other hand, that is the grim reality across the region, so in one sense, it is a realistic test of on-the-ground policy outcomes, in the world as it really is. Moreover, Bruno et al. (2018) found that MPAs globally / generally, regardless of enforcement, failed to affect resistance to or recovery from large-scale disturbances (discussed in detail here: https://theseamonster.blog/2019/01/bruno-et-al-2019-arms-supp/).

Regardless, we agree with the reviewer's general point and have added relevant text to the Discussion on this issue: it is explained more here in the text, "Enforcement of the protected areas in our study varied among sites and over time from 'good' (regular patrols, poaching is minimum, as in Hol Chan) to 'inadequate' (irregular patrols, poaching exists, as in South Water) (Table S1)" (L. 406-408).

And as for factors influencing coral cover, I don't know what to think of the HII metric. With the rapid development of resorts on the islands along the BBR during the study period, increases in cruise ships, live-aboards, etc., it's not clear how the HII dataset truly represents anthropogenic impacts. I'm not arguing against the analysis, just that it's easy to poke holes in this data set, and the authors may want to acknowledge its limitations.

Please refer to the discussion of HII below.

On the topic of broad-scale stressors, the authors emphasize heat waves and climate change (L88), sometimes linking this with disease (L73), and often not (L353). ***In fact, there is no conclusive link between heat stress events and disease events, and the latter may be the result of anthropogenic introductions (e.g., ballast water),*** flare-ups of virulent disease

variants, etc. This is important because these two things (climate, disease) are distinctly different, and this paper vaguely merges them together.

We believe there is substantial evidence that ocean heating has played a significant role in some observed coral disease outbreaks, including yellow band and white band in the Caribbean and white syndrome in the Pacific (Selig et al. 2006, Bruno et al. 2007, Harvell et al. 2009, Randall and van Woesik 2015, Howells et al. 2020). Moreover, global warming has been widely implicated (with strong, but perhaps not "conclusive" supporting evidence) in the uptick in wildlife diseases in terrestrial and marine environments (Harvell et al. 1999, 2002). We don't disagree with the idea that pathogen introduction is also important. In fact, it's more often than not changes in all three aspects of the disease triangle—host, pathogen, and environment—that trigger outbreaks, as discussed in (Bruno 2015). 6/11/2021 12:38:00 AM

In our view, like bleaching, changes in colony growth and fecundity, increased storm damage, etc, some (not all) coral diseases are at least exacerbated by ocean heating. They could also be affected by local impacts like nutrient pollution (Bruno et al. 2003), and we have added text noting this possibility (L. 76-83).

Further, the data in this manuscript **are now 5 years old**, and in that time SCTLD has ravaged reefs across the Caribbean.

True!

It is likely that, even if there had been no decline in coral cover over the study period, it would have begun dropping precipitously from SCTLD in the past 2 years, and plunged below 10% without warming events playing any role. Interestingly, SCTLD stops spreading under warm conditions, suggesting it is not tied to warm water events. So, how does SCTLD play into this paper's recommendations (L50-52)?

We agree. This is a great suggestion and we have added text to the Introduction and Discussion on these points (L. 69–74 and 359–361).

What is the point of MPAs and reduced emissions when reef-building corals are functionally (reproductively) extinct on the vast majority of Caribbean reefs? Are studies such as these for the history books, or to guide us as Indo-Pacific reefs increasingly progress in the same way as those in the Caribbean?

No-take MPAs (if they are well-designed, old, large, enforced, etc.) can have massive positive effects on harvested reef taxa. We disagree that it is already game-over for corals, although, there's no doubt things are looking rather bleak.

It was surprising that the authors didn't look more closely at macroalgal cover as a function of MPA status. Was seaweed cover higher inside or outside of MPAs (at any one time point, over several, etc)? This variable has been targeted in many other studies, both

because seaweeds grow rapidly and overfishing removes herbivores. Looking at the red and blue dots in Fig 2, there doesn't seem to be a consistent pattern for macroalgae across the time-series, but it would be interesting to know if there is an effect of MPAs (despite the lack of MPA validation).

We agree! We should have discussed changes in algal cover, over time, with protection, etc. We have added text to the Methods, Results, and Discussion and this new graphic:

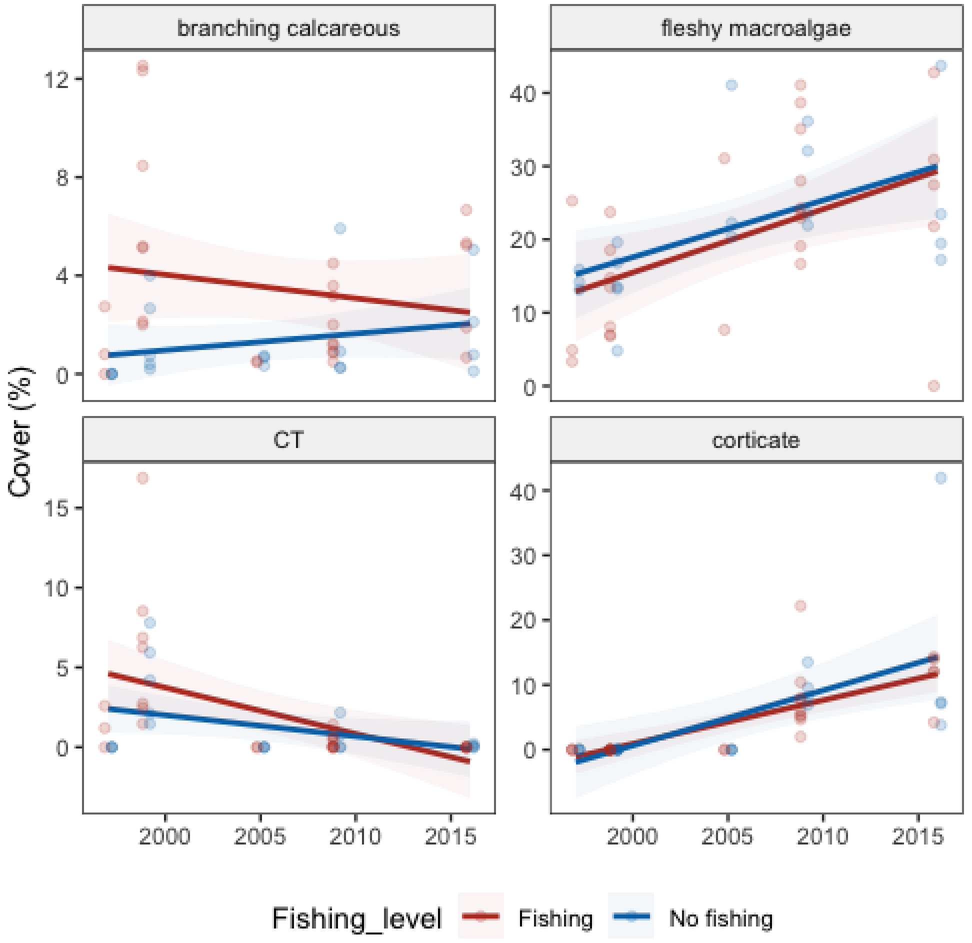

I may have missed reference to this, but were the authors planning on providing their raw data somewhere? I didn't see a link in their SI.

Indeed! All of the study data and code are already freely available without restriction here: https://github.com/calves06/Belizean_Barrier_Reef_Change

Reviewer #3: Overall, the paper is good, and I support publication. The comments below are to help the editor/authors consider various specific points. A few points are more confusing than others and need to be more clearly addressed.

Line 78. Please provide direct refs for “nutrient loading” as a secondary driver. I think this is controversial although I’ve not checked recent literature, and believe it needs a little more “play” in this introd.

We added the following text to address this concern: “On local scales, increased sedimentation and pollution from coastal development affect coral reefs by smothering corals and increasing turbidity (28,29). Secondary drivers of coral degradation include factors that have increased the cover of fleshy macroalgae (seaweeds), such as the death of scleractinian corals and the consequent opening of space and other resources (30); nutrient loading; and reduced herbivory. Herbivory has declined primarily because of the loss of the black sea urchin *Diadema antillarum* due to a regional disease outbreak (31) and severe reductions of populations of herbivorous fishes due to fishing” (32–37).

Line 81-83. I don’t disagree that the crux is about relative importance (local vs regional/global). This is key and I’m glad your paper is focusing on this. But, it seems to me a that looking for various ‘direct effects’ is missing a big point, that there could be ultimate causes (climate, for ex) and then secondary causes related to the ultimate by INDIRECT effects. A conceptual model addressing this would be a good addition if links are supported enough to be causal, but this is really a relatively small point re: your paper.

The conceptual model is a very interesting idea but is beyond the scope of this paper. However, our approach would detect an indirect effect since we are simply testing for spatial-temporal associations between the benthic components and the putative drivers.

Line 130-131. I know what you mean, but you might could say it more clearly or directly. You swam a 30 m long transect over a period of 5-7 min (depending on swimmer, and conditions I suppose….) and took photo…etc. Is less convoluted in description.

Fair point. We’ve rewritten it as: “At each site, dive teams laid out four to ten, 25–30 m x 2 m belt transects down the centers of reef spurs, perpendicular to the shoreline. The transects generally began on or near the shoulders of the spurs at 15–18 m depth, shoreward of the drop-off that characterizes most of the reefs, and ran upward toward the reef crest. Transects were parallel to each other and were usually separated by >10 m. Divers worked in buddy pairs, in which one diver laid out the transect tape and the other used a digital camera in an underwater housing to obtain videos or still-frame images of the benthos. At each site, we photographed or videotaped the belt transects at a standard distance of 25 cm above the benthos, using a bar projecting from the front of the camera housing to maintain distance from the bottom. In all sampling years except 2016, we obtained underwater videos along the belt transects and extracted still frames from those videos (as outlined below). In 2016, we photographed the transects using a GoPro HERO4 by swimming at a rate of 5–7 minutes along the 30-m-long transect and taking a photograph every five seconds.” (L. 125–137).

Lines 172-174. "We extracted HII values for the BBR (Fig. S1) and calculated the sum of the HII 173 scores of grid cells within a 50-km, 75-km, and 100-km buffer from the center-coordinates of 174 each study site (Table S1). We used HII scores within the 50-km buffer for the final analysis…" This bothers me some because the smallest scale was the one chosen, which presumably showed interesting results. But, the values are down to 1 km2 scale, so it would seem as if smaller scales should have been investigated as well. Large scales might unduly weight against the local factors that you argue against later. If this isn't the case, you need to MAKE the case!

The point of using a 50-km "buffer distance" from each site is to account for cumulative human impacts within that distance, and because it was the most complete across all study sites. Using a smaller buffer is possible of course, but for some sites the HII will be 0 because there is no mainland or island to extract information from. We did test 25-km and 10-km buffers but had that issue with those buffers (see figure below). Several sites have 0 HII values under 10 km because they are far from any land or caye (e.g., Pompian, Halfmoon Key) with HII data. Please see below for a visualization of HII at all study sites and across the 10, 25, 50, 75, and 100 km buffers.

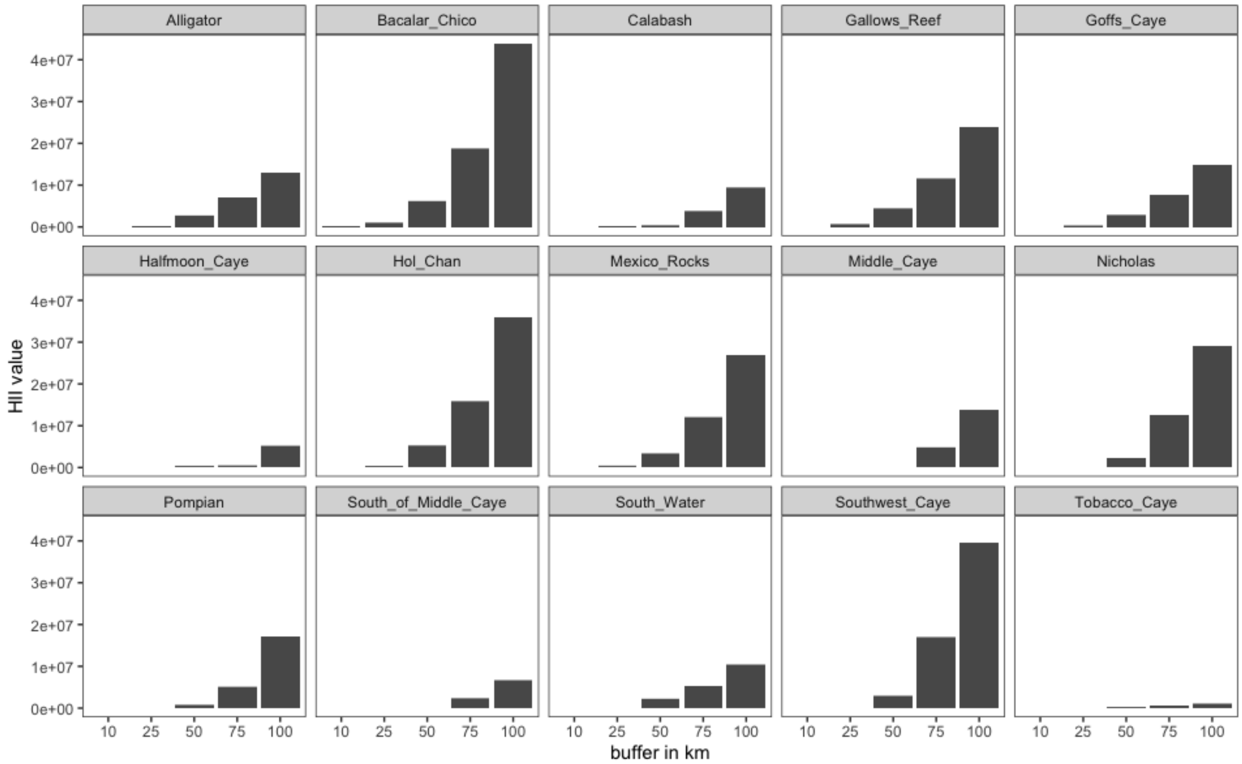

Lines 184-185. “Other studies have found that TSA Freq is a significant predictor 185 of coral-cover loss and coral-disease prevalence (62–64).” Ok, but is it a good predictor or a weak one? R2 or measures of effect size needed since I’ve (and other readers) won’t have read 62-64.

We include the R2 of the Bayesian generalized linear mixed model that predicts live coral cover (see Table 2). The best model had year and TSA Freq as significant predictors of changes in live coral cover. The model explains ~67% of the variability observed in coral cover accounting for fixed and random effects. Not bad for ecology!

Line 193. “…logit-transformed percent covers of key benthic categories.” Which were what????? You go on to list predictors in some detail but not response. Is this coral cover? By Species? By Genera? By functional group? Etc, etc. more detail please, after all it’s your RESPONSE variables!

Good point. We’ve clarified in the text: “The response variables were the logit-transformed site- and year-specific percent covers of five key benthic categories (hard corals, macroalgae, CTB, gorgonians, and sponges), several coral taxa (coral groups, genera or species), and three major macroalgal functional groups (calcareous, fleshy, and corticate) (Table S2).” (L. 209–212).

Lines 196-7. “…blme prior with a wishart distribution was imposed over 197 the covariance of the random effect and modeled coefficients”. Why Wishart? Were any others tried? Give me a reason to accept that this is a good analysis, with some ways to back this up and let me go check on my own if I wish.

During exploratory models we tried a blme prior with three different distributions: ‘wishart’, ‘gamma’, and ‘null’. We did not try any inverse distribution options such as ‘invwishart’ or ‘invgamma’ as they do not make sense for our data. In all exploratory models, different prior distributions did not significantly improve model performance (e.g., similar AICs). As such, we decided to use a wishart distribution as it is the default in the blmer function.

Lines 197-8. “All predictor variables were 198 additive…” I assume because you CHOSE not to test for interactions? Is that correct? If so, say so, and defend why.

We did test for several interactions in exploratory models (e.g., Table S4), but they all resulted to be insignificant and thus we removed it from final models. Additive explanatory variables resulted in models with the lowest AICs. We provided further clarity on this issue in the text: “In exploratory analysis we modeled the interaction between TSA Freq and protection status as well as TSA Freq and HII; however, these interactions did not improve model fit and were not significant, so we dropped from the models (see R code in supplementary information)” (L. 218–221). We also explained further here: Local protection (within MPAs) or geographic isolation from local impacts (sites with low HII

scores) did not reduce the effect of ocean-temperature anomalies on these four affected coral taxa: the TSA Freq*HII and TSA Freq*Protection Status interaction terms were not significant (see R Code in supplementary information and Table S4). In fact, the model structures with the interaction terms generally performed worse (they had higher AIC scores) than the additive models, and thus these interaction terms were dropped from the final models" (L. 268–274).

Lines 200-208. So…it looks like that Year is the factor relating to climate change. Is that right?

That is not correct. Our best performing models have Year (centered) as a numerical explanatory variable to account for the temporal ("repeated measures") nature of the study and *to test for change in benthic community structure over time*. But year is not correlated with TSA freq (vif < 1.2). Thus, our interpretation is that coral cover declined over time and part of that decline was due to thermal stress anomalies.

But TSA freq is also in the model, are they not correlated and thus measuring some of the same variance in your response??? TSA freq would seem to increase with year, does it not? Need to discuss this to quite the drums….

No. Like essentially all measures of ocean warming and climate change more broadly, TSA freq does not increase annually, which is to say in a clean, linear way. This is because so many natural factors, like ENSO, affect temperature (and also rainfall, storm intensity, etc.). Over a longer period of time—e.g., 30 plus years—indeed it would increase, but again not in a year-on-year manner.

In this study, for 10 sites, TSA freq *was actually greatest in 1999* (see Table S2 from our Supplement below), following the extremely warm summer of 1998.

Also note that TSA freq is both year- and site-specific (also see Fig S2). Within a given year, thermal stress varies substantially among sites and we are not using a single, regional measure of thermal stress each year.

S2 Table Variation in three metrics of thermal stress anomaly (TSA) across years and sites. Darker color represents higher values within each TSA metric. TSA is the weekly SST minus the maximum weekly climatological SST. The frequency of TSA (TSA_Freq) is the number of instances TSA is over 1 degree Celsius over the previous 52 weeks [1]. The frequency of historical TSA (TSA_Freq_hist) is the number of times since the beginning of the dataset (1982) that TSA was >1 degree Celsius. The frequency of TSA between survey years (TSA_Freq_btw_surveys) is the number of instances since the previous survey year that TSA was >1 degree Celsius [1].

Site	TSA_Freq					TSA_Freq_hist					TSA_Freq_btw_surveys				
	1997	1999	2005	2009	2016	1997	1999	2005	2009	2016	1997	1999	2005	2009	2016
Alligator	1	3	1	5	2	9	15	22	33	52	4	3	5	6	14
Bacalar Chico	2	4	2	1	5	14	19	25	31	54	7	4	5	3	20
Calabash	0	1	0	4	3	24	25	34	42	54	9	1	4	5	10
Gallows Reef	1	2	1	2	0	16	20	24	37	57	6	2	4	5	16
Goffs Caye	0	3	1	4	1	11	15	19	30	43	4	3	3	6	11
Halfmoon Caye	1	2	1	1	1	6	8	18	28	47	2	2	6	6	15
Hol Chan	1	2	2	3	3	19	22	28	39	53	6	2	6	6	11
Mexico Rocks	0	3	3	4	0	7	12	22	31	40	4	3	9	5	5
Middle Caye	0	3	1	1	2	13	16	26	37	60	4	3	7	6	20
Nicholas	1	5	0	1	2	14	20	28	39	69	6	5	6	6	25
Pompian	1	5	0	3	2	19	25	31	43	67	7	5	3	7	21
South of Middle Caye	0	4	3	1	2	9	13	24	36	59	3	4	8	8	20
South Water	1	3	0	0	3	15	18	26	35	55	4	3	5	4	17
Southwest Caye	0	4	3	1	2	9	13	24	36	59	3	4	8	8	20
Tacklebox	1	5	4	4	5	27	33	45	56	72	7	5	11	5	13

Lines 263-264. "….ordination analysis, there were major compositional shifts in the dominant benthic 264 assemblages during 1997–2005 (left) and 2009–2016 (right) at every site (Fig. 6, Table 3), 265 supporting the results of our models." Please explain more the L and R aspect of this and how it supports the results of your model. Also, I presume you mean that "support the results of your model" means that you posed certain models (additive only…but ok) a priori and that this is what is supported. It might be more clear to earlier say your models are posed as a priori hypotheses, and later discuss your results in light of the HYPOTHESES rather than relating directly to MODELS. A small point, actually.

Thank you for pointing this out. We removed language referring to hypotheses, and have clarified in the results of the ordinations in the manuscript: "There were major compositional shifts in the dominant benthic assemblages during 1997–2005 (left) and 2009–2016 (right) at every site (Fig. 7, Table 3). The PERMANOVA showed that, among all covariates, time explained about 50% of the variability in benthic community changes ($F = 45.8$, $p < 0.001$) and was the only significant predictor of change in overall community composition (Fig. 7, Table 3). Protection status, HII, and TSA frequency combined only accounted for 6% of community differences and were not good predictors of overall change of all taxa studied (Table 3). In 1997–2005, the benthic communities of the BBR were dominated by CTB and long-lived,

massive reef-building corals such as *Orbicella* spp. and *C. natans*. During 2009–2016, composition had shifted to domination by small and/or weedy hard-coral species, macroalgae, and gorgonians (Fig. 7)" (L. 309–318).

Discussion: lines 308-318. I "get" what you're saying but your analysis + explanation leaves me a little cold. First, you have temp (TSA freq) which you 'bought in to ' as a global factor. **But then it has little if any effect. But, time does….so basically you seem to transfer your time argument from TSA to time per se.**

TSA (ocean temperature) *was* significantly inversely related to the cover of several key reef taxa: *Acropora* spp. *Orbicella* spp., *Colpophyllia natans*, and other coral species (Figs. 3 and 4; Table S4).

We understand the remark about transferring the time, which is based on Reviewer 2's incorrect interpretation of the role of time and thermal stress in the study and statistical model. Year is simply the effect that indicates change over time, whereas TSA freq (and the other predictors) attempts to "explain" some fraction of that observed variance

If I've missed the mark here, then it means your explanations at several points need revising and expanding…because that's the way it seems to me. I guess the point is: if you have TSA freq which is presumable increasing over time, why have time in the model too??? I suspect there's a good reason, but I didn't see it in the text.

See above. Also, we clarified this in the text (lines 311–318). Thank you for pointing out this issue. We see why it is confusing.

Reviewer #4: This is a significant amount of reef data collected over a long time period. However, can such bold statements about the inutility of local regulations be based on 15 survey sites that are 30x2m wide? Coral reefs have such diverse environmental conditions and influencers of health. Here are my comments that the authors should address:

1. You cannot estimate local impacts with global datasets - there is a clear scale mismatch and you cannot infer local community status.

Reviewer 4's point here is unclear. You can measure local impacts with global data, including remote-sensing data. For example, reef ecologists have for decades measured ocean temperature remotely to predict (with a high degree of accuracy) local bleaching events, disease outbreaks, coral loss, etc. (Aronson et al. 2002, Bruno et al. 2007, Hughes et al. 2017, 2018, Stuart-Smith et al. 2018). Also see the large body of work by Josh Cinner's and Tim McClanahan's groups that have used other global datasets that describe local human impacts (e.g., the global 'gravity' index of fishing pressure) to predict and understand reef fish community dynamics (e.g., Cinner et al. 2018). Numerous studies have found satellite-based measurements of ocean surface temperature are remarkably good at predicting fine-grained

benthic temperature measurements made with instruments on the reef (e.g., Selig et al. 2006). Below is a graph from a 20-year old paper (Bruno et al. 2001) demonstrating this point.

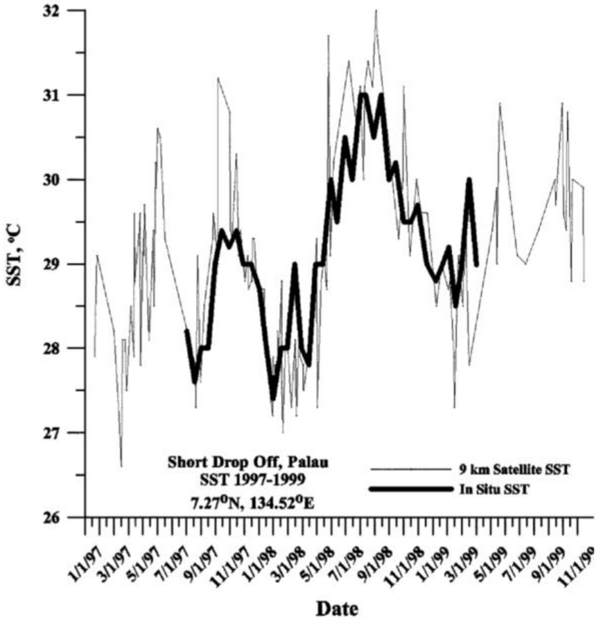

Fig. 3 SST data at the Short Drop Off site in Palau for 1997–1999. In situ measurements were made by hand and 9-km night-time satellite SST data are from the NASA/JPL AVHRR Oceans Pathfinder program

2. There was no investigation into the quality of the HII dataset which can underrepresent human impacts at the local scale, especially in less developed countries since many of the inputs are remote sensing-based and cannot be detected with coarse resolution data.

We are unaware of information supporting this argument. The most important component of HII (human population density) is not measured remotely; it is based on national on-the-ground census data (via the Statistical Institute of Belize: https://sib.org.bz/publications/census-reports/).

The purpose of the HII is to “map anthropogenic impacts on the environment that can be used in conservation planning, natural resource management and research on human-environment interactions.” The dataset is global at 1-km grid-cell resolution that combines nine global data layers including human population density, human land use and infrastructure (e.g., built-up areas, nighttime lights, land use/land cover), and human access (e.g., coastlines, roads, railroads, navigable rivers). The dataset is produced by the Wildlife Conservation Society (WCS) and the Columbia University Center for International Earth Science Information Network (CIESIN).

We have added substantial text to the Discussion to better explain what HII is and how we used it, e.g., lines 444-476 in which we also include some important caveats about our use of the metric.

Was there any vetting of the data with local experts to see if the changes detected in the HII approximated reality?

Absolutely. Our co-authors include two Belizeans (Castillo and Bood) and arguably Belize's three leading reef conservation and ecology experts (McField, Castillo, and Bood). Four of our coauthors work for NGOs focused on the BBR (McField, Bood, Cox and Valdivia), a majority of us completed our doctoral research on the BRR, and two of us have worked on the BBR intensively for nearly 30 years (McField and Aronson). In short, we are the local and international experts. We did examine the HII scores (below) and they do reflect the widely perceived geographic variance in human development and local human impacts.

For example, as expected (depicted in Fig. S1) HII is high around Belize City, much lower in southern Belize, and high in Honduras. 50-km HII at the sites near Belize City (where the local impacts including murky water flowing out of the Belize river are obvious) including Gallows and Alligator are very high (41029 and 37768 respectively). HII values for sites near the rapidly developing San Pedro island resorts are also high (Table S1). In contrast, the geographically isolated sites, where water clarity is far better and local impacts from the major human developments of the mainland should be relatively lower, have far lower HII values (e.g., 100 and 2480 for Middle and Halfmoon cayes respectively).

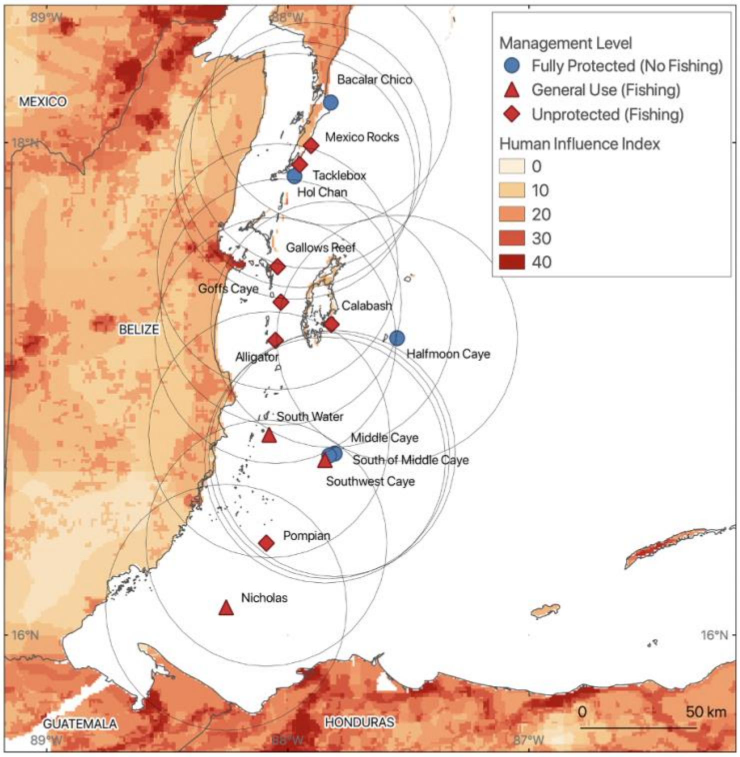

The year-to-year changes need to be vetted with locals. HII is purely land-based and many of the sites are on islands that are not accurately detected with HII, are far from the coastline, thus limiting influence. HII is a land-based model and ocean sites also require a marine-based threat model to properly determine impacts.

It is land-based and purposefully so. It is meant as an indicator of location-specific, land-based "stressors" that could affect reef organisms and influence the community dynamics measured in this longitudinal study: primarily pollution, including sediments from land-use change/coastal development, nutrients, herbicides etc. In theory, the influence of these stressors that originate onshore (or at the land-sea interface) should dissipate with isolation from their source (assuming they are "point-source"). As you can see in the HII map above, HII is mapped on the islands, no matter how small.

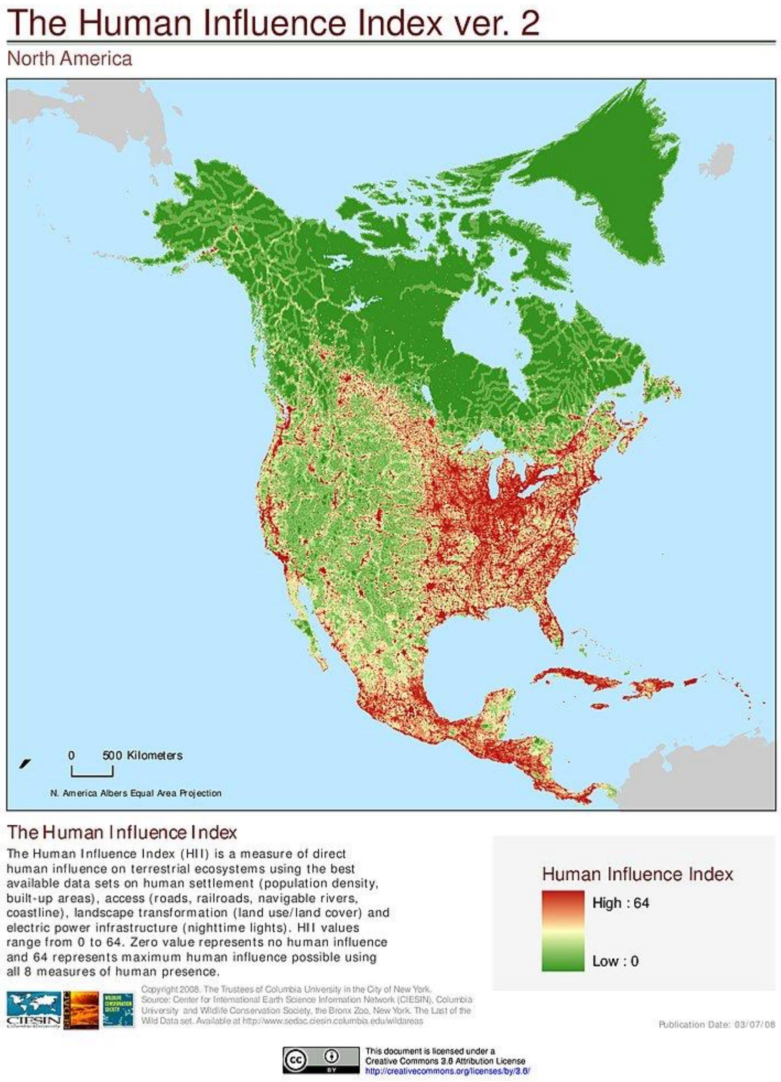

All that said, **we fully recognize the limitations of this measure of local human impacts.** It does not, for example, include dredging near San Pedro (and elsewhere) to expand islands for development. It does not include potential impacts of cruise ships not anchored at ports. We wish it was better. We wish direct, *in situ* measurements of the stressors (e.g., sediment and nutrient pollution) that result from the coastal development measured by HII at our sites were available, but they aren't in Belize or nearly anywhere on earth. That's part of the reason so little is known about the role of local impacts: there are very few programs that monitor them. Next to no data are available to relate to observed changes in reef communities. Tracking these factors (nutrients, sediment, etc.) at each of our sites continuously for two decades would have been ideal, but there's no funding mechanism to do this.

That said, ***we do have excellent data on the proximate causes of these potential stressors:*** the human population centers (human density), land-use changes, ports, industrial complexes, roads etc: the human footprint, which is what HII measures. We have fine-grained data on these metrics of the human footprint and we use their combined scoring (HII) to ask whether local human development is related to benthic community dynamics. If local development (and its resulting physical and chemical stressors) harmed some taxa and / or benefited others, you'd predict that these effects would be stronger in closer proximity to it (people, ports, deforestation, etc). Reefs isolated by tens of kilometers should be less or not-at-all affected. We didn't find that. That is not so to say there aren't any effects of humanity's terrestrial footprint on the adjacent coral reefs; just that we weren't able to detect it. That is probably because local impacts have been swamped by the much larger, more obvious, and more easily detectable effects of ocean warming, disease, large storms, etc. This interpretation is concordant with most studies that have looked at both local and large-scale impacts (e.g., Bruno and Valdivia 2016, for fish: Valdivia et al 2017, Mora 2008)

3. Since there were many storms and hurricanes over the study time period, these can act as a catalyst for breaking up corals which can colonize elsewhere and stimulate growth. It is difficult to detect the changes in coral cover across the reef if you are going back to the same survey transects and only looking at the same 30x2m area.

This point is unclear to us. Repeatedly sampling the same location / transects is a much more powerful way to detect change than randomly selecting locations each year (although the latter approach also has the benefit of increased generalizability). In addition, coral species that are easily broken up and propagated by storms (e.g., branching corals such as *Acroporas*) had very low live cover during the study period.

4. There are many papers that demonstrate MPAs have a positive effect on coral cover. (Strain et al 2019; Strain et al 2019; Mellin et al 2016;Magdaong et al 2014; Selig and Bruno 2010;Rogers 2009).

Some papers do indeed demonstrate that MPAs can potentially benefit coral cover, *but not in response to large scale disturbances like storms, disease outbreaks and marine heatwaves*. A

recent review and meta-analysis on the topic (Bruno et al. 2018), found 18 studies that measured coral resistance to and/or recovery from large-scale disturbances in 66 MPAs and 89 unprotected control sites (Table 1 below). The study outcomes were remarkably consistent: they found no significant effect of protection on total coral cover loss (11 out of 11 studies) or on the rate of post-disturbance coral-cover gain (15 out of 16 studies). The mean decline in absolute coral cover averaged across studies immediately after a disturbance tended to be greater, not smaller, inside MPAs [12.3% ± 5.5% (mean ± 1 SE)] than it was on unmanaged reefs (4.5% ± 2.0%; paired t-test, $t = -1.987$, d.f. = 10, $p = 0.08$). There was no difference in coral recovery rates between control and managed sites (paired t-test, $t = -0.814$, d.f. = 14, $p = 0.43$).

Table 1 Characteristics and outcomes of 18 tests of the managed-resilience concept

Reference	Location	MPA effect?	Sites (MPA/control)	Duration (years)	Disturbance[a]
Bégin et al. 2016	Saint Lucia, Caribbean	No	6/6	10	S
Bood 2006	Belize, Caribbean	No	3/3	8	B, C
Coelho & Manfrino 2007	Little Cayman, Caribbean	No	2/5	6	B, C, D
Darling et al. 2010	Kenya	No	3/4	21	B
Graham et al. 2008	Indian Ocean	No	9/10	10	B
Graham et al. 2015	Seychelles	No	4/12	17	B
Halpern et al. 2013	Solomon Islands	No	3/3	5	S
Harris et al. 2014	Seychelles	No	6/15	6	B
Huntington et al. 2011	Belize, Caribbean	No	1/1	10	B, C, D
Jones et al. 2004	Papua New Guinea	No	4/4	7	B, S
Manfrino et al. 2013	Little Cayman, Caribbean	No	2/4	13	B, S
McClanahan 2008	Kenya	No	3/4	12	B
McClanahan et al. 2001	Kenya	No	4/4	1	B
Miller et al. 2009	US Virgin Islands, Caribbean	No	5/1	2	B, D
Mumby & Harborne 2010	Bahamas, Caribbean	Yes	4/6	3	B, C
Muthiga 2009	Kenya	No	2/2	12	B
Russ et al. 2015	Philippines	No	2/2	30	B
Toth et al. 2014	Florida Keys	No	3/3	15	B, C, D

We considered only direct empirical field tests that measured change in absolute coral cover within at least one protected area [marine protected areas (MPAs) and fully protected or no-take marine reserves] and one control area. We searched for articles using the terms "coral reef + resilience OR recovery OR resistance" via Web of Science. The tests measured resistance to (as change in coral cover before versus immediately after a disturbance) and/or recovery from (as rate of coral cover change over time after a disturbance) disturbances. The MPAs come from 15 countries (and two regions of the United States—the Florida Keys and the US Virgin Islands) and a wide range of reef types and biogeographic realms. The average study duration was 10 years. One study (Bood 2006) was an unpublished MS thesis.

[a]B, bleaching or warming event; C, cyclone or hurricane; D, disease; S, sediment.

Note that most papers cited by the reviewer demonstrate that MPA have a weak positive effect on total coral cover under very specific conditions. Below are some examples.

Strain et al 2019: The authors found that only old (>10 yro), well-enforced, no-take MPAs had higher coral cover than fished sites driven by the cover of massive coral growth forms (see Fig. 2 in the paper). They also found that both direct (+0.06) and indirect (+0.4) effects of no-take MPAs on coral cover were weak (see Fig. 3 in the paper).

Mellin et al 2016: Although the authors found marginally greater stability within MPAs upon disturbances compared to non-MPAs (*t*-test, $p = 0.048$), the loss of live coral was similar (*t*-test, $p > 0.05$) in MPAs and non-MPAs. In fact, the authors found significantly higher loss of live coral cover in MPAs than in non-MPAs following diseases (see Fig. S1 in the paper). Mellin et al. (2016), however, found that despite a decline in coral cover across the Great Barrier Reef since 1994, MPAs have helped slow the recent decline in coral cover.

Magdaong et al 2014: They found a net positive effect of no-take MPAs on coral cover over a period of three decades (1981–2010) across the Philippines, where coral cover was higher in fully protected MPAs than in non-MPAs. However, this was due to the prohibition of destructive fishing practices (explosives) and direct mechanical damage (anchoring) within fully protected MPAs. As they explained in the discussion. "*This is most likely due to protection which helps in the improvement of the overall coral reef health by reducing anthropogenic pressure (primarily fishing, eliminating destructive fishing and anchor damage) that affects the substrate condition*"

Selig and Bruno 2010: In this global analysis of over 300 MPAs, Selig and Bruno suggested that older MPAs help maintain coral cover.

Rogers 2009: If this citation refers to the comments published by Caroline Rogers in RSPB in response to Mora 2008, this note does not address positive or negative effects of MPAs on coral reefs.

Did the authors look at management effectiveness for these parks and were there surveys carried out for the MPAs? Do they think that since an MPA is in place, it is actually preventing people from fishing? MPAs will not work if enforcement and management is not carried out.

We did in Cox et al. (2017). We measured fish composition and biomass inside no-take reserves and in nearby control sites and found they were (largely) ineffective. We could not quantify fishing activity in the no-take reserves, but we obtained enforcement information for the no-take reserves starting in 2011 based on eco-audits conducted by the Healthy Reef Initiative group. There is no reliable enforcement information in most of these no-take reserves before 2011 We agree this is an issue, and we address it above in response to a similar query from reviewer 2.

5. While reducing emissions is the number one priority, the authors should acknowledge that saving coral reefs will require a number of local actions including better watershed and fisheries management, restoration of coastal habitats, and active restoration that focuses on restoring the

natural recovery processes and genotypes that are the most resistant to disease and bleaching. You cannot dismiss the importance of local regulations (reserves, reduced fishing pressure, limiting development) which have been shown to have a positive effect on reefs.

Fair points. We have revised the conclusions to state: “However, environmental changes caused by local human activities, such as increased nutrient concentrations, are not monitored in Belize, making it challenging to directly assess their effects. The rapid elimination of global greenhouse emissions is clearly paramount for the survival and recovery of the BBR. However, we also urge local authorities to increase resources to support the enforcement of existing MPAs and to mitigate any possible effects of coastal development on Belize’s coral reefs.” (L. 484–490).

General comments: The length of study and observations contained in this manuscript is unusual. The findings are placed in the context of long term shifts in coral ecosystems related to far field effects such as bleaching (warming temperatures) disease (warming temperature) and hurricane damage. The data collection and methods are sound (although I question whether you might need a balanced design to test hypotheses such as fishing or not fishing) and the results are presented in this context of far field causes of community composition shifts. The independent variables tested were fishing or not, year, index of Human activity and deviation of ocean heatwave events (TSA freq). The results indicate significant trends and correlations with year and with TSA. If you only look at the results, the changes are occurring over a 20 year period. The causes are not.

The authors spend much of the discussion on disease, bleaching and hurricane damage, all of which are not contained in the measurements made

We measured ocean temperature, which is a known driver of the severity of these and other large-scale disturbances.

except for the changes in percent cover. It would have been nice in hindsight to measure losses during bleaching events, document the disease, and use BACI for hurricane damage.

We tend to agree, but numerous other studies have done this already and published their results (Table 1), so it would also have been redundant.

There is no solution to this problem other than the authors were not able to measure independent variables needed over a 20 year period to explain why the shift in composition has occurred. The approach to explain the changes in composition using HII and TSA seem logical but probably too course for the relatively few number of sites spread out over a large area.

We disagree. We did measure five of the variables believed to drive changes in coral community composition: temperature, coastal development (HII), local protection in this study, and macroalgal cover and fish composition and biomass in Cox et al (2017).

The resolution of TSA freq (temperature) and HII (local impacts) is actually quite fine-grained: 1-km^2 for HII, and ~4-km^2 for TSA, which is the highest resolution covering the longest time period of any satellite-based ocean dataset.

Otherwise, temperature anomalies would more strongly explain bleaching events mentioned in the discussion? The reader has to accept assumptions that are laid out throughout the introduction and in the discussion. However, the assumptions are well supported by existing knowledge, and the conclusions are reasonable given the stat of knowledge in the field. Ln 308 and associated paragraph is a good example. The data and analysis, as presented, were not designed to test whether MPAs prevent reef degradation;

The study was specifically designed to test this hypothesis; see coauthor Bood's MS thesis, based on the surveys in 2006 of six of the study sites:

Bood N D. Recovery and resilience of coral assemblages on managed and unmanaged reefs in Belize: A long-term study. [Master's Thesis]. Mobile, AL, University of South Alabama; 2006.

rather the study shows a trend of coral losses at all sites and increase in macroalgae cover. The community change data and the management strategy to prevent coral loss has not worked. However, it is only clear from existing knowledge that some direct and indirect effects come from MPA establishment and that a majority of studies have found that MPAs are not working to slow or prevent the decline of reef-building reefs.

This statement supports the conclusion of our study and contradicts the previous statement that "the data and analysis, as presented, were not designed to test whether MPAs prevent reef degradation."

Ln 102-104. Should this information be in the introduction?

We were tempted and went back and forth about this. It feels more like background information and a recognition of prior related efforts, but we'd be happy to reconsider and move to the Introduction if the editor prefers.

Ln 104 Not clear what sites are sampled and when sampled. 3 sites were sampled every year; were the other sites randomized through time or was this more opportunistic based on projects specific to the sites. it is a combination of different data sets from the different projects, how were biases about what sites were sampled in each year reconciled?

No. This study was originated in 1997 by coauthor McField as a single, comprehensive assessment of the state of Belize's reefs to focus on 6 sites. The project then was expanded the following year as a long-term monitoring study with more sites (See Table S1). Due to funding limitations and other logistical constraints (many co-authors needing to teach during the

academic year), we were not able to access every site every year (or every sampling trip). From the pool of sites, those selected on a given trip were somewhat haphazard, often depending on weather and safety concerns, although we always sampled close to an equal number of protected and unprotected sites each year.

The statistical model would not necessarily be a 'balanced' model.

No it wasn't. But that is not an assumption of the analysis or this type of study.

Ln 113 SCUBA- spell out acronyms when used for first time

We believe SCUBA is such a common acronym that it is not necessary to spell it out.

Ln 129 Was the GOPRO camera fitted with the 25 cm rod? GOPROs have fisheye type lenses which change the analysis area.

Yes it was. We added this detail to the methods: "At each site, we photographed or videotaped the belt transects at a standard distance of 25 cm above the benthos, using a bar projecting from the front of the camera housing to maintain distance from the bottom." (L. 132–134).

Ln 160 Does Coral Point Count automatically convert image-level point count to percent cover? Please explain the conversion

No it does not. Conversion is accomplished by dividing the number of points over each benthic component by the total number of points recorded for a given image.

Fig. S1; The scale of the map makes it easier to see the values on the mainland but more difficult in the islands where sampling was conducted. The table S1 has the raw values but is there any way to see the color ramp at the sampling sites? I can't think of a way other than providing a paneled figure with fewer sites zoomed in to see the color ramp of the HII scores. It is supplemental information

Thank you for this excellent suggestion. We adjusted Figure 1 to include zoomed-in panels for the following four main regions of offshore sites: Ambergris Caye, Turneffe Atoll, Glovers Reef Atoll, and Sapodilla Cayes.

Ln 202; I'm not as familiar with GLMMs as with GLMs but this seems to be a reasonable approach; however I'm still concerned about the sampling sites and years not being the same except for 3 sites. Is this type of analysis robust to imbalances? It would seem that changing

human index and temperature index would be year dependent while fishing and non fishing would be constant.

The Human Influence Index is site-specific but it is static and does not change over time. That is, each site is associated with a unique HII that is based on 9 human-related impact layers within 50 km of each site and calculated over a 10-year period (1994–2005). TSA Freq varies for each site and for each sampling year. Protection vs no protection is constant

Ln 229 I think it is commendable that the authors shared the code and processed data on github.

Thank you!

Ln 265- “supporting the results of our models” this should go in the discussion not results. Figures and supporting information is fine.

OK.

Literature Cited

Aronson, R. B., W. F. Precht, M. A. Toscano, and K. H. Koltes. 2002. The 1998 bleaching event and its aftermath on a coral reef in Belize. Marine Biology 141:435–447.

Bruno, J. F. 2015. Marine biology: The coral disease triangle. Nature Climate Change 5:302–303.

Bruno, J. F., I. M. Côté, and L. T. Toth. 2018. Climate Change, Coral Loss, and the Curious Case of the Parrotfish Paradigm: Why Don’t Marine Protected Areas Improve Reef Resilience?307–334.

Bruno, J. F., L. E. Petes, C. D. Harvell, and A. Hettinger. 2003. Nutrient enrichment can increase the severity of coral diseases. Ecology Letters 6:1056–1061.

Bruno, J. F., E. R. Selig, K. S. Casey, C. A. Page, B. L. Willis, C. D. Harvell, H. Sweatman, and A. M. Melendy. 2007. Thermal stress and coral cover as drivers of coral disease outbreaks. PLoS Biology 5:e124.

Bruno, J. F., C. E. Siddon, J. D. Witman, P. L. Colin, and M. A. Toscano. 2001. El Niño related

coral bleaching in Palau, Western Caroline Islands. Coral Reefs 20:127–136.

Bruno, J. F., and A. Valdivia. 2016. Coral reef degradation is not correlated with local human population density. Scientific Reports 6:29778.

Cinner, J. E., E. Maire, C. Huchery, M. A. MacNeil, N. A. J. Graham, C. Mora, T. R. McClanahan, M. L. Barnes, J. N. Kittinger, C. C. Hicks, S. D'Agata, A. S. Hoey, G. G. Gurney, D. A. Feary, I. D. Williams, M. Kulbicki, L. Vigliola, L. Wantiez, G. J. Edgar, R. D. Stuart-Smith, S. A. Sandin, A. Green, M. J. Hardt, M. Beger, A. M. Friedlander, S. K. Wilson, E. Brokovich, A. J. Brooks, J. J. Cruz-Motta, D. J. Booth, P. Chabanet, C. Gough, M. Tupper, S. C. A. Ferse, U. R. Sumaila, S. Pardede, and D. Mouillot. 2018. Gravity of human impacts mediates coral reef conservation gains. Proceedings of the National Academy of Sciences 115:E6116–E6125.

Cox, C., A. Valdivia, M. McField, K. Castillo, and J. Bruno. 2017. Establishment of marine protected areas alone does not restore coral reef communities in Belize. Marine Ecology Progress Series 563:65–79.

Gill, D. A., M. B. Mascia, G. N. Ahmadia, L. Glew, S. E. Lester, M. Barnes, I. Craigie, E. S. Darling, C. M. Free, J. Geldmann, S. Holst, O. P. Jensen, A. T. White, X. Basurto, L. Coad, R. D. Gates, G. Guannel, P. J. Mumby, H. Thomas, S. Whitmee, S. Woodley, and H. E. Fox. 2017. Capacity shortfalls hinder the performance of marine protected areas globally. Nature 543:665–669.

Harvell, C. D., S. Altizer, I. M. Cattadori, L. Harrington, and E. Weil. 2009. Climate change and wildlife diseases: when does the host matter the most? Ecology 90:912–920.

Harvell, C. D., K. Kim, J. M. Burkholder, R. R. Colwell, P. R. Epstein, D. J. Grimes, E. E. Hofmann, E. K. Lipp, A. Osterhaus, R. M. Overstreet, J. W. Porter, G. W. Smith, and G. R. Vasta. 1999. Emerging marine diseases - climate links and anthropogenic factors. Science 285:1505–1510.

Harvell, C. D., C. E. Mitchell, J. R. Ward, S. Altizer, A. P. Dobson, R. S. Ostfeld, and M. D.

Samuel. 2002. Climate warming and disease risks for terrestrial and marine biota. Science 296:2158–2162.

Howells, E. J., G. O. Vaughan, T. M. Work, J. A. Burt, and D. Abrego. 2020. Annual outbreaks of coral disease coincide with extreme seasonal warming. Coral Reefs 39:771–781.

Hughes, T. P., K. D. Anderson, S. R. Connolly, S. F. Heron, J. T. Kerry, J. M. Lough, A. H. Baird, J. K. Baum, M. L. Berumen, T. C. Bridge, D. C. Claar, C. M. Eakin, J. P. Gilmour, N. A. J. Graham, H. Harrison, J.-P. A. Hobbs, A. S. Hoey, M. Hoogenboom, R. J. Lowe, M. T. McCulloch, J. M. Pandolfi, M. Pratchett, V. Schoepf, G. Torda, and S. K. Wilson. 2018. Spatial and temporal patterns of mass bleaching of corals in the Anthropocene. Science 359:80.

Hughes, T. P., J. T. Kerry, M. Álvarez-Noriega, J. G. Álvarez-Romero, K. D. Anderson, A. H. Baird, R. C. Babcock, M. Beger, D. R. Bellwood, R. Berkelmans, T. C. Bridge, I. R. Butler, M. Byrne, N. E. Cantin, S. Comeau, S. R. Connolly, G. S. Cumming, S. J. Dalton, G. Diaz-Pulido, C. M. Eakin, W. F. Figueira, J. P. Gilmour, H. B. Harrison, S. F. Heron, A. S. Hoey, J.-P. A. Hobbs, M. O. Hoogenboom, E. V. Kennedy, C. Kuo, J. M. Lough, R. J. Lowe, G. Liu, M. T. McCulloch, H. A. Malcolm, M. J. McWilliam, J. M. Pandolfi, R. J. Pears, M. S. Pratchett, V. Schoepf, T. Simpson, W. J. Skirving, B. Sommer, G. Torda, D. R. Wachenfeld, B. L. Willis, and S. K. Wilson. 2017. Global warming and recurrent mass bleaching of corals. Nature 543:373–377.

Randall, C. J., and R. van Woesik. 2015. Contemporary white-band disease in the Caribbean has been driven by climate change. Nature Climate Change 5:375–379.

Selig, E. R., C. D. Harvell, J. F. Bruno, B. L. Willis, C. A. Page, K. S. Casey, and H. Sweatman. 2006. Analyzing the relationship between ocean temperature anomalies and coral disease outbreaks at broad spatial scales. Pages 111–128 Coral reefs and climate change: science and management. American Geophysical Union, Washington, DC.

Stuart-Smith, R. D., C. J. Brown, D. M. Ceccarelli, and G. J. Edgar. 2018. Ecosystem

restructuring along the Great Barrier Reef following mass coral bleaching. Nature 560:92–96.

Valdivia, A., C. E. Cox, and J. F. Bruno. 2017. Predatory fish depletion and recovery potential on Caribbean reefs. Science Advances 3:e1601303.
