# Supplementary material for: Twenty years of change in benthic communities across the Belizean Barrier Reef
Source: PLoS One. 2022 Jan 18;17(1):e0249155. doi: 10.1371/journal.pone.0249155 (PMC8765652; doi:10.1371/journal.pone.0249155)
Supplement: Attachment — Submitted filename: Review notes PONE-D-21_07319 reviewer 1.docx [file pone.0249155.s002.docx]

TITLE: Twenty years of change in benthic communities across the Belizean Barrier Reef

Authors: Alves et al.

General comments: The length of study and observations contained in this manuscript is unusual. The findings are placed in the context of long term shifts in coral ecosystems related to far field effects such as bleaching (warming temperatures) disease (warming temperature) and hurricane damage. The data collection and methods are sound (although I question whether you might need a balanced design to test hypotheses such as fishing or not fishing) and the results are presented in this context of far field causes of community composition shifts. The independent variables tested were fishing or not, year, index of Human activity and deviation of ocean heatwave events (TSA freq). The results indicate significant trends and correlations with year and with TSA. If you only look at the results, the changes are occurring over a 20 year period. The causes are not. The authors spend much of the discussion on disease, bleaching and hurricane damage, all of which are not contained in the measurements made except for the changes in percent cover. It would have been nice in hindsight to measure losses during bleaching events, document the disease, and use BACI for hurricane damage. There is no solution to this problem other than the authors were not able to measure independent variables needed over a 20 year period to explain why the shift in composition has occurred. The approach to explain the changes in composition using HII and TSA seem logical but probably too course for the relatively few number of sites spread out over a large area. Otherwise, temperature anomalies would more strongly explain bleaching events mentioned in the discussion? The reader has to accept assumptions that are laid out throughout the introduction and in the discussion. However, the assumptions are well supported by existing knowledge, and the conclusions are reasonable given the stat of knowledge in the field. Ln 308 and associated paragraph is a good example. The data and analysis, as presented, were not designed to test whether MPAs prevent reef degradation; rather the study shows a trend of coral losses at all sites and increase in macroalgae cover. The community change data and the management strategy to prevent coral loss has not worked. However, it is only clear from existing knowledge that some direct and indirect effects come from MPA establishment and that a majority of studies have found that MPAs are not working to slow or prevent the decline of reef-building reefs.

Ln 102-104. Should this information be in the introduction?

Ln 104 Not clear what sites are sampled and when sampled. 3 sites were sampled every year; were the other sites randomized through time or was this more opportunistic based on projects specific to the sites. If it is a combination of different data sets from the different projects, how were biases about what sites were sampled in each year reconciled? The statistical model would not necessarily be a ‘balanced’ model.

Ln 113 SCUBA- spell out acroyms when used for first time

Ln 129 Was the GOPRO camera fitted with the 25 cm rod? GOPROs have fisheye type lenses which change the analysis area.

Ln 160 Does Coral Point Count automatically convert image-level point count to percent cover? Please explain the conversion

Fig. S1; The scale of the map makes it easier to see the values on the mainland but more difficult in the islands where sampling was conducted. The table S1 has the raw values but is there any way to see the color ramp at the sampling sites? I can’t think of a way other than providing a paneled figure with fewer sites zoomed in to see the color ramp of the HII scores. It is supplemental information

Ln 202; I’m not as familiar with GLMMs as with GLMs but this seems to be a reasonable approach; however I’m still concerned about the sampling sites and years not being the same except for 3 sites. Is this type of analysis robust to imbalances? It would seem that changing human index and temperature index would be year dependent while fishing and non fishing would be constant.

Ln 229 I think it is commendable that the authors shared the code and processed data on github.

Ln 265- “supporting the results of our models” this should go in the discussion not results.

Figures and supporting information is fine.
